# Common variation near *ROBO2* is associated with expressive vocabulary in infancy

**DOI:** 10.1038/ncomms5831

**Published:** 2014-09-16

**Authors:** Beate St Pourcain, Rolieke A.M. Cents, Andrew J.O. Whitehouse, Claire M.A. Haworth, Oliver S.P. Davis, Paul F. O’Reilly, Susan Roulstone, Yvonne Wren, Qi W. Ang, Fleur P. Velders, David M. Evans, John P. Kemp, Nicole M. Warrington, Laura Miller, Nicholas J. Timpson, Susan M. Ring, Frank C. Verhulst, Albert Hofman, Fernando Rivadeneira, Emma L. Meaburn, Thomas S. Price, Philip S. Dale, Demetris Pillas, Anneli Yliherva, Alina Rodriguez, Jean Golding, Vincent W.V. Jaddoe, Marjo-Riitta Jarvelin, Robert Plomin, Craig E. Pennell, Henning Tiemeier, George Davey Smith

**Affiliations:** 1 Medical Research Council Integrative Epidemiology Unit, University of Bristol, Oakfield House, 15-23 Oakfield Grove, Bristol BS8 2BN, UK; 2 School of Oral and Dental Sciences, University of Bristol, Lower Maudlin Street, Bristol BS1 2LY, UK; 3 School of Experimental Psychology, University of Bristol, 12a Priory Road, Bristol BS8 1TU, UK; 4 Generation R Study Group, Erasmus MC-University Medical Centre, Postbus 2040, 3000 CA Rotterdam, The Netherlands; 5 Department of Child and Adolescent Psychiatry/Psychology, Erasmus MC-University Medical Centre, Postbus 2060, 3000 CB Rotterdam, The Netherlands; 6 Telethon Kids Institute, Centre for Child Health Research, University of Western Australia, 100 Roberts Road, Subiaco, Western Australia 6008, Australia; 7 Department of Psychology, University of Warwick, Coventry CV4 7AL, UK; 8 Medical Research Council, Social, Genetic and Developmental Psychiatry Centre, Institute of Psychiatry, King’s College London, De Crespigny Park, Denmark Hill, London SE5 8AF, UK; 9 Department of Genetics, Evolution and Environment, UCL, UCL Genetics Institute, Darwin Building, Gower Street, London WC1E 6BT, UK; 10 Department of Epidemiology and Biostatistics, Medical Research Council (MRC) Public Health England (PHE) Centre for Environment and Health, School of Public Health, Imperial College London, Norfolk Place, London W2 1PG, UK; 11 Bristol Speech and Language Therapy Research Unit, University of the West of England, Frenchay Hospital, Frenchay Park Road, BS16 1LE Bristol, UK; 12 School of Women’s and Infants’ Health, University of Western Australia, 374 Bagot Road, Subiaco, Western Australia 6008, Australia; 13 School of Social and Community Medicine, University of Bristol, Canynge Hall, 39 Whatley Road, Bristol BS8 2PS, UK; 14 University of Queensland Diamantina Institute, Translational Research Institute, University of Queensland, 37 Kent Street Woolloongabba, Queensland 4102, Australia; 15 Department of Epidemiology, Erasmus MC-University Medical Centre, Postbus 2040, 3000 CA Rotterdam, The Netherlands; 16 Department of Internal Medicine, Erasmus MC-University Medical Centre, Postbus 2040, 3000 CA Rotterdam, The Netherlands; 17 Department of Psychological Sciences, Birkbeck, University of London, Malet Street, London WC1E 7HX, UK; 18 Institute for Translational Medicine and Therapeutics, University of Pennsylvania School of Medicine, 3400 Civic Center Boulevard, Building 421, Philadelphia, Pennsylvania 19104-5158, USA; 19 Department of Speech and Hearing Sciences, University of New Mexico, 1700 Lomas Boulevard NE Suite 1300, Albuquerque, New Mexico 87131, USA; 20 Faculty of Humanities, Logopedics, Child Language Research Center, University of Oulu, BOX 1000, Oulu 90014, Finland; 21 Mid Sweden University Department for Psychology/Mittuniversitetet Avdelningen för psykologi, 83125 Östersund, Sweden; 22 Department of Pediatrics, Erasmus MC-University Medical Centre, Postbus 2060, 3000 CB Rotterdam, The Netherlands; 23 Unit of Primary Care, Oulu University Hospital, Kajaanintie 50, PO Box 20, FI-90220, Oulu 90029, Finland; 24 Department of Children and Young People and Families, National Institute for Health and Welfare, Aapistie 1, Box 310, FI-90101 Oulu, Finland; 25 Institute of Health Sciences, University of Oulu, PO Box 5000, Oulu FI-90014, Finland; 26 Biocenter Oulu, University of Oulu, PO Box 5000, Aapistie 5A, OuluFI-90014, Finland; 27 These authors contributed equally to this work

## Abstract

Twin studies suggest that expressive vocabulary at ~24 months is modestly heritable. However, the genes influencing this early linguistic phenotype are unknown. Here we conduct a genome-wide screen and follow-up study of expressive vocabulary in toddlers of European descent from up to four studies of the EArly Genetics and Lifecourse Epidemiology consortium, analysing an early (15–18 months, ‘one-word stage’, *N*_Total_=8,889) and a later (24–30 months, ‘two-word stage’, *N*_Total_=10,819) phase of language acquisition. For the early phase, one single-nucleotide polymorphism (rs7642482) at 3p12.3 near *ROBO2*, encoding a conserved axon-binding receptor, reaches the genome-wide significance level (*P*=1.3 × 10^−8^) in the combined sample. This association links language-related common genetic variation in the general population to a potential autism susceptibility locus and a linkage region for dyslexia, speech-sound disorder and reading. The contribution of common genetic influences is, although modest, supported by genome-wide complex trait analysis (meta-GCTA *h*^2^_15–18-months_=0.13, meta-GCTA *h*^2^_24–30-months_=0.14) and in concordance with additional twin analysis (5,733 pairs of European descent, *h*^2^_24-months_=0.20).

## Abstract (web summary)

The genetic basis of expressive vocabulary in children around 2 years old is poorly understood. Here, the authors show that a genetic variant near the *ROBO2* gene is associated with early language acquisition in the general population and highlight a potential genetic link between language-related common genetic variation and a linkage region for dyslexia, speech-sound disorder and reading.

## Graphical abstract

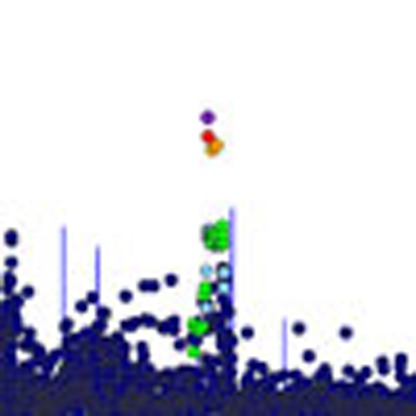

The number of distinct spoken words is a widely used measure of early language abilities, which manifests during infancy^1^. Word comprehension (known as receptive language) in typically developing children starts at the age of about 6–9 months^2^, and the spontaneous production of words (known as expressive language) emerges at about 10–15 months^1^,^3^. During the next months the accumulation of words is typically slow, but then followed by an increase in rate, often quite sharp, around 14–22 months of age (‘vocabulary spurt’)^1^,^4^. As development progresses, linguistic proficiency becomes more advanced, with two-word combinations (18–24 months of age)^1^,^3^ and more complex grammatical structures (24–36 months of age)^1^,^3^ arising, accompanied by the steady increase in vocabulary size. Expressive vocabulary is therefore considered to be a rapidly changing phenotype, especially between 12 and 24 months^5^, with zero size at birth, ~50 words at 15–18 months^1^,^3^, ~200 words at 18–30 months^1^,^3^, ~14,000 words at 6 years of age^3^,^4^ and ≥50,000 words in high school graduates^6^,^7^.

Twin analyses of cross-sectional data suggest that expressive vocabulary at ~24 months is modestly heritable (*h*^2^=0.16–0.38)^8^,^9^, and longitudinal twin analyses have reported an increase in heritability of language-related factors during development (*h*^2^=0.47–0.63, ≥7 years of age)^10^. Large-scale investigations of common genetic variation underlying growth in language skills, however, are challenging owing to the complexity and varying nature of the phenotype. This is coupled with a change in psychological instruments, which are used to assess these abilities with progressing age. Current genome-wide association studies (GWASs) using cross-sectional data on language abilities in childhood and adolescence have failed to identify robust signals of genome-wide association^11^,^12^, and genes influencing earlier, less-complex linguistic phenotypes are currently unknown.

To attempt to understand genetic factors involved in language development during infancy and early childhood, we perform a GWAS and follow-up study of expressive vocabulary scores in independent children of European descent from the general population and analyse an early (‘one-word stage’) and a later (‘two-word stage’) phase of language acquisition. We report a novel locus near *ROBO2*, encoding a conserved axon-binding receptor, as associated with expressive vocabulary during the early ‘one-word’ phase at the genome-wide significance level, and provide heritability estimates for expressive vocabulary during infancy and early childhood.

## Results

### Genome-wide association analyses

**Figure 1:**
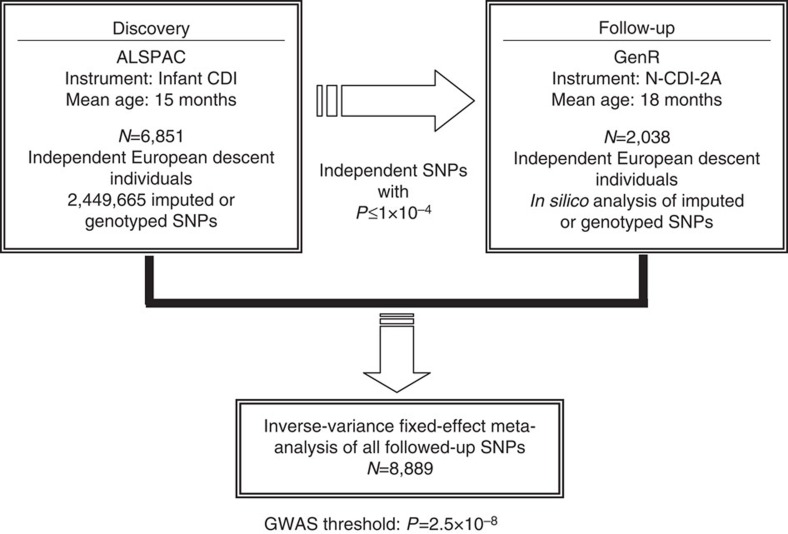
Study design for the genome-wide screen of early expressive vocabulary. Expressive vocabulary between 15 and 18 months of age was assessed using different forms of the MacArthur Communicative Development Inventories (CDI). Detailed phenotype descriptions are given in Supplementary Data 1.

**Figure 2:**
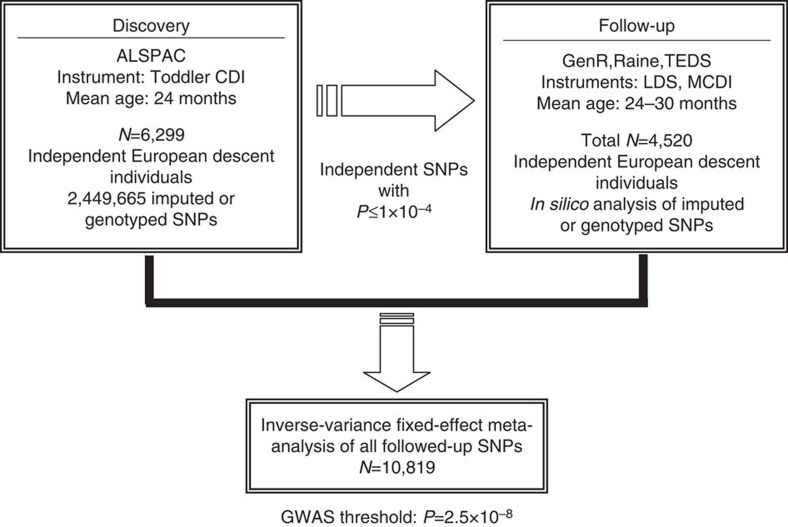
Study design for the genome-wide screen of later expressive vocabulary. Expressive vocabulary between 24 and 30 months of age was assessed with different forms of the MacArthur Communicative Development Inventories (CDI) and the Language Development Survey (LDS). Detailed phenotype descriptions are given in Supplementary Data 1.

We conducted two cross-sectional genome-wide screens corresponding to an early (15–18 months, *N*_Total_=8,889) and a later (24–30 months, *N*_Total_=10,819) phase of language acquisition, respectively, each adopting a two-stage design (Figs 1 and 2; Supplementary Data 1). During these developmental phases, expressive vocabulary was captured with age-specific word lists (adaptations of the MacArthur Communicative Development Inventories (CDI)^13^,^14^,^15^,^16^,^17^ and the Language Development Survey (LDS)^18^, Methods). However, measures of expressive vocabulary were not normally distributed and differed in their symmetry (Supplementary Data 1; Supplementary Fig. 1), and association analysis was therefore carried out using rank-transformed scores (Methods). Within the discovery cohort, a total of 2,449,665 autosomal genotyped or imputed single-nucleotide polymorphisms (SNPs) were studied in 6,851 15-month-old and 6,299 24-month-old English-speaking toddlers, respectively. Genome-wide plots of the association signals are provided in Supplementary Figs 2 and 3. For the early phase, the strongest association signal was observed at rs7642482 on chromosome 3p12.3 near *ROBO2* (*P*=9.5 × 10^−7^, Supplementary Table 1) and for the late phase at rs11742977 on chromosome 5q22.1 within *CAMK4* (*P*=3.5 × 10^−7^, Supplementary Table 2). All independent variants from the discovery analysis (associated *P*≤10^−4^, Supplementary Tables 1 and 2), including these SNPs, were taken forward to a follow-up study (Methods). This included 2,038 18-month-old Dutch-speaking children for the early phase and 4,520 24–30-month-old Dutch or English-speaking children for the later phase (Supplementary Data 1).

**Figure 3:**
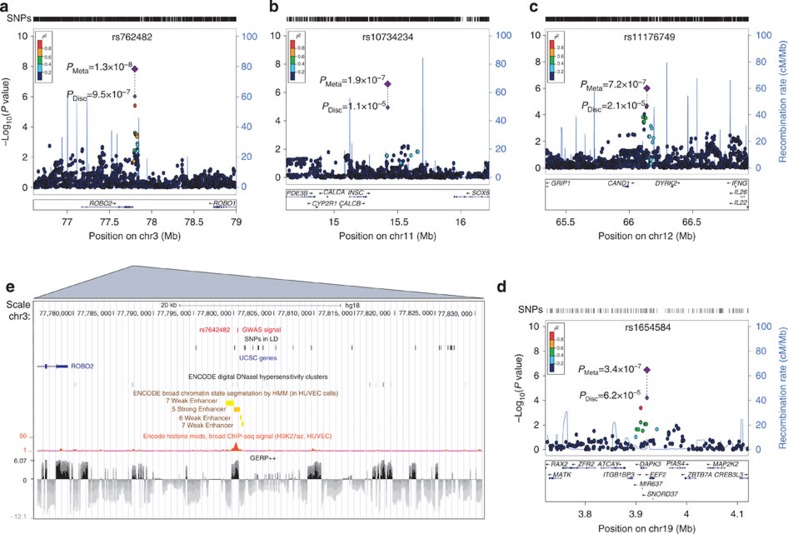
Association plots for early expressive vocabulary signals. For the 3p12.3 (**a**), 11p15.2 (**b**), 12q15 (**c**) and 19p13.3 (**d**) region, SNPs are plotted with their discovery -log_10_ *P* value as a function of the genomic position (hg18). *P* values were generated from weighted linear regression of the rank-transformed vocabulary score (15–18 months of age) on allele dosage. *P* values of discovery SNPs taken forward to the follow-up analysis are denoted by a small purple diamond (*P*_Disc_) and their combined meta-analysis *P* value (*P*_Meta_) is represented by a large purple diamond. The local linkage disequilibrium (LD) structure near the associated region is reflected by recombination rates estimated from Hapmap CEU (phase II). SNPs are coloured on the basis of their correlation with the lead signal (based on pairwise LD*r*^2^ values). (**e**) Detailed annotation of the genomic region at 3p12.3 using the UCSC Genome Browser (hg18) including rs7642482 and SNPs in LD (±500 kb, LD*r*^2^>0.3, Hapmap). Tracks for ENCODE digital DNaseI hypersensitivity clusters, ENCODE histone modifications and chromatin state segmentation in umbilical vein endothelial cells (HUVEC), as well as Genomic Evolutionary Rate Profiling (GERP++) scores (lifted from hg19) are included.

For four independent loci from the early phase GWAS (rs7642482, rs10734234, rs11176749 and rs1654584), but none for the later phase analysis, we found evidence for association within the follow-up cohort (*P*<0.05), assuming the same direction of effect as in the discovery sample (Table 1; Supplementary Tables 1–4). In the combined analysis of all available samples (Table 1; Fig. 3a–d) rs7642482 on chromosome 3p12.3 near *ROBO2* (the strongest signal in the discovery cohort) reached the genome-wide significance level (*P*=1.3 × 10^−8^), and the three other signals approached the suggestive level (rs10734234 on chromosome 11p15.2 near *INSC*, *P*=1.9 × 10^−7^; rs11176749 on chromosome 12q15 near *CAND1*; *P*=7.2 × 10^−7^ and rs1654584 on chromosome 19p13.3 within *DAPK3*; *P*=3.4 × 10^−7^).

Each of these four polymorphisms explained only a small proportion of the phenotypic variance (adjusted regression R^2^: for rs7642482=0.34–0.35%, rs10734234=0.27–0.35%, rs11176749=0.25–0.27% and rs1654584=0.22–0.49%) in both the discovery and the follow-up cohort, but together the four SNPs accounted for >1% of the variation in early expressive vocabulary scores (joint adjusted regression *R*^2^=1.10–1.45%). For the SNP reaching genome-wide significance, rs7642482, each increase in the minor G-allele was associated with lower expressive vocabulary, although, due to the rank-transformation, an interpretation of the magnitude of the genetic effect is not informative. An empirical estimate of the genetic effect in the discovery sample, suggested a decrease of 0.098 s.d. in expressive vocabulary scores (95% confidence interval: 0.058; 0.14) per increase in G-allele. We are aware, however, that this signal might be prone to the ‘winner’s curse’ (that is, an overestimation of the effect) and requires further replication within independent samples.

### Characterization of the lead association signals

rs7642482 is located ~19 kb 3′ of *ROBO2* (OMIM: 602431), which encodes the human roundabout axon guidance receptor homologue 2 (*Drosophila*) gene. An *in silico* search for potentially functional effects using the University of California Santa Cruz Genome Browser^19^ provided no evidence that rs7642482 or proxy SNPs (*r*^2^>0.3) relate to protein-coding variation within *ROBO2*. For this, we also confirmed the observed linkage disequilibrium structure within the discovery cohort through local imputation of chromosome 3 using the 1,000 Genomes reference panel (v3.20101123, Supplementary Fig. 4). The sequence at rs7642482 and the flanking genomic interval are, however, highly conserved (rs7642482 Genomic Evolutionary Rate Profiling (GERP)^20^ score=3.49; regional average GERP score near rs7642482 (derived from 100 bases surrounding rs7642482, GWAVA^21^)=3.06; average GERP score for coding sequences^20^ >2). Encyclopaedia of DNA elements (ENCODE)^22^ data indicate that in umbilical vein endothelial cells (HUVEC), rs7642482 overlaps with regulatory chromatin states, such as H3K27ac^23^,^24^, which are predicted to be a strong enhancer^25^ (Fig. 3e). Additional searches using HaploReg (v2) (ref. 26)^26^ identified overlaps with further regulatory DNA features, such as DNase I hypersensitive sites and binding sites for transcription factors (lrx, Pou3f2_1). This suggests that variation at rs7642482 might be implicated within regulatory mechanisms in embryonic cell types, consistent with a peak of *ROBO2* expression in the human brain during the first trimester (Supplementary Fig. 5). There was no evidence for *cis* expression quantitative trait loci (eQTL) within ±1 Mb of rs7642482 in postnatally derived cell types or adult brain tissue, based on searches of public eQTL databases (seeQTL)^27^,^28^.

Since little is known about the genetic factors affecting language acquisition, the ‘suggestive’ signals at 11p15.2, 12q15 and 19p13.3 may also stimulate future research. rs10734234 resides within the vicinity of *INSC* (197 kb 3′ of the gene), encoding an adaptor protein for cell polarity proteins (OMIM: 610668). rs11176749 is located near *CAND1* (144 kb 3′ of the gene) encoding a F-box protein-exchange factor (OMIM: 607727), which regulates the ubiquitination of target proteins, and rs1654584 is an intronic SNP within *DAPK3* encoding the death-associated protein kinase 3, which plays a key role in apoptosis (OMIM: 603289).

Within a further step, we investigated whether the reported association signals are influenced by potential covariates, such as gestational age^29^ and maternal education^30^. These have been previously linked to late language emergence in infancy^29^ and the total number of spoken words in early childhood^30^, respectively. Studying up to 8,889 15–18-month-old children from the discovery and follow-up cohort, the association signal at rs7642482 increased when gestational age was adjusted for (adjusted *P*_meta_=4.0 × 10^−9^, 0.36–0.38% explained variance), while adjustment for maternal education did not affect the association (Supplementary Tables 5 and 6). For the remaining SNPs, there was little or no effect on the strength of the genetic association when these covariates were controlled for.

To explore whether the reported association signals influence linguistic skills other than early-phase expressive vocabulary, we also investigated a series of language-related measures during development. We observed no evidence for association between the four SNPs and first single-word utterances in 4,969 12-month-old Finnish children (Supplementary Data 1; Supplementary Table 7). However, this age pertains to a developmental stage where expressive vocabulary is very low, that is, the majority of children speak about one or two words, and pre-linguistic communication skills are still developing^31^. All early-phase signals were furthermore attenuated or even abolished when investigated for association with word-production scores during the later phase of language acquisition (24–30 months, Supplementary Fig. 6). This age band spans a phase where growth in linguistic proficiency may relate more to early grammar development including two-word combinations^1^, than a vocabulary of single words. Overall, the phenotypic correlations between early and later expressive vocabulary scores were moderate within cohorts with multiple linguistic measures (0.48<*ρ*≤0.57, Supplementary Data 1), and evidence for genetic correlations, based on genome-wide complex trait analysis (GCTA)^32^,^33^, was mixed (Avon Longitudinal Study of Parents and Children (ALSPAC): *r*_g_(s.e.)=0.69(0.20), *P*=0.02), Generation R Study (GenR): *r*_g_(s.e.)=−0.32(0.97), *P*=0.18). There was also no association between the four reported SNPs and other language-related cognitive outcomes, including verbal intelligence scores, in middle childhood (8–10 years of age) when studying up to 5,540 children from the discovery cohort, apart from nominal associations with reading speed (rs7642482 *P*=0.009; rs1654584 *P*=0.0035; Supplementary Tables 8 and 9). Thus, the observed genetic associations, especially at rs7642482, are likely to be time-sensitive and specific to the early phase of language acquisition.

### Twin analysis and GCTA

A twin study of 5,733 twin pairs of European descent, including a subset of children from the follow-up cohorts, supported the (modest) influence of additive genetic effects on variability in expressive vocabulary at ~24 months (*a*^2^(s.e.)=0.20(0.008); Table 2; Supplementary Tables 10 and 11, Methods) and was consistent with previous reports on a smaller sample^9^. Estimates from twin analysis and GCTA^32^, performed on the discovery sample, were furthermore in close concordance (ALSPAC GCTA *h*^2^(s.e.)_15-months_=0.13(0.05); GCTA *h*^2^(s.e.)_24-months_=0.17(0.06); Table 2). However, in the smaller-sized follow-up samples, GCTA heritability, especially for the later phase, was close to zero (Table 2), and is likely to reflect impaired power during the follow-up. Combining GCTA heritability estimates using meta-analysis techniques (Methods), provided similar estimates as observed for the discovery cohort alone (meta-GCTA *h*^2^(s.e.)_15–18-months_=0.13(0.05), meta-GCTA *h*^2^(s.e.)_24–30 months_=0.14(0.05)).

## Discussion

This study reports a genome-wide screen and follow-up study of expressive vocabulary scores in up to 10,819 toddlers of European origin investigating an early phase (15–18 months) and a later phase (24–30 months) of language acquisition. On the basis of the combined analysis of all available samples, our study identifies a novel locus near *ROBO2* as associated with expressive vocabulary during the early phase of language acquisition.

Robo receptors and their Slit ligands (secreted chemorepellent proteins) are highly conserved from fly to human^34^,^35^ and play a key role in axon guidance and cell migration. In vertebrates, Robo2 is involved in midline commissural axon guidance^36^, the proliferation of central nervous system progenitors^37^, the spatial positioning of spiral ganglion neurons^38^ and the assembly of the trigeminal ganglion^39^, which is the sensory ganglion of the trigeminal nerve. The latter is particularly important for speech production in humans^40^, as the trigeminal nerve provides motor supply to the muscles of mastication, which control the movement of the mandibles, and in addition the nerve transmits sensory information from the face. Thus, genetic variation at *ROBO2* may be linked to both speech production abilities and expressive vocabulary size within children of the general population.

Rare recurrent *ROBO2* deletions have been discovered in patients with autism spectrum disorder^41^, a severe childhood neuro-developmental condition where core symptoms include deficits in social communication^42^, and decreased *ROBO2* expression has been observed in the anterior cingulate cortex^43^ and in lymphocytes of individuals with autism^44^. Indeed, the 3p12-p13 region has been linked to dyslexia^45^, and quantitative dyslexia traits^46^, as well as quantitative speech-sound disorder traits and reading^47^. The dyslexia linkage findings^45^ have been related to a specific SNP haplotype within *ROBO1*^48^, a neighbouring gene of *ROBO2.* In animal models, *Robo1* and *Robo2* are mostly co-expressed and it has been shown that both receptors function cooperatively, for example, with respect to the guidance of most forebrain projections^49^. Thus, it is possible that variation within both *ROBO1* and *ROBO2* might also contribute to the linkage signals within the reported regions, and our findings highlight *ROBO2* as a novel, not yet investigated candidate locus.

Common polymorphisms within *ROBO1* have also been associated with reading disability^50^ and with performance on tasks of non-word repetition^51^, which is related to phonological short-term memory deficits. However, none of these previously reported *ROBO1* variants (rs12495133, rs331142, rs4535189 and rs6803202)^50^,^51^ were associated with early word production scores within our study (Supplementary Table 12). Vice versa, we also found no association between rs7642482 (*ROBO2*) and language-related measures, including phonological memory and verbal intelligence in middle childhood, nor was there any association with expressive vocabulary during the later phase of language acquisition (24–30 months of age) or with very first single-word utterances at about 12 months of age. Instead, our findings suggest that the identified *ROBO2* signal is specific for an early developmental stage of language acquisition (15–18 months of age), which is characterized by a slow accumulation of single words, followed by an increase in rate that is sometimes related to a ‘vocabulary spurt’^1^,^4^. Both *in silico* analyses and the increase in signal after adjustment for gestational age support the hypothesis that expressive vocabulary during this phase may be affected by perinatal or early postnatal gene regulatory mechanisms. It is furthermore possible that the enhancer effect predicted within HUVEC also relates to a yet uncharacterized embryonic cell type, where expression changes are only detectable on the single-cell level. For example, during the trigeminal ganglion formation placode/neural crest cells travel as individual cells to the site of ganglion formation, and *Robo2* appears to be expressed in discrete, dispersed regions in the surface ectoderm^39^. This is characteristic of cells, which are about to detach and migrate^39^. Thus, it will require further molecular studies to characterize the biological mechanisms underlying the observed *ROBO2* association in more detail.

In line with previous findings^8^,^9^, estimates from twin analysis and GCTA (based on large samples) suggest that the proportion of phenotypic variation in early expressive vocabulary, which is attributable to genetic factors, is modest. The concordance of twin and large-sample GCTA heritability estimates indicates, however, that most of this genetic variation is common and that there is little ‘missing heritability’. Thus, a large proportion of common genetic variation influencing early expressive vocabulary might be captured by current GWAS designs, given sufficient power.

To conclude, this study describes genome-wide association between rs7642482 near *ROBO2* and expressive vocabulary during an early phase of language acquisition where children typically communicate with single words only. The signal is specific to this developmental stage, strengthened after adjustment for gestational age, and links overall language-related common genetic variation in the general population to a potential autism susceptibility locus as well as a linkage region for dyslexia, speech-sound disorder and reading on chromosome 3p12-p13.

## Methods

### Phenotype selection and study design

Consistent with the developmental pattern of language acquisition, the analysis of children’s expressive vocabulary in infancy was divided between an early phase (15–18 months of age, Fig. 1) and a later phase (24–30 months of age, Fig. 2) and conducted using independent individuals of up to four population-based European studies with both quantitative expressive vocabulary scores and genotypes available (early phase: total *N*=8,889; later phase: total *N*=10,819).

Expressive vocabulary scores were measured with age-specific-defined word lists and either ascertained with adaptations of the MacArthur CDI^13^,^14^,^15^,^16^,^17^ or the LDS^18^ and based on parent-report. The CDIs were developed to assess the typical course and variability in communicative development in children of the normal population (8–30 months of age)^13^. The LDS was designed as a screening tool for the identification of language delay in 2-year-old children^18^. Both measures have sufficient internal consistency, test-retest reliability and validity^18^,^52^,^53^.

Expressive vocabulary during the early phase was captured by an abbreviated version of the MacArthur CDI (Infant Version^13^, 8–16 months of age, Supplementary Data 1) within the discovery cohort (ALSPAC, *N*=6,851, Supplementary Fig. 1a). Note, the Infant CDI has recently become also known as CDI Words and Gestures^54^. A Dutch adaptation of the short-form version of the MacArthur CDI (N-CDI 2A)^14^,^16^ was used within the follow-up cohort (GenR, *N*=2,038). Scores in both cohorts comprised both expressive and receptive language aspects (‘says and understands’) and showed a positively skewed data distribution (1.95<skewness≤2.39; Supplementary Data 1).

Vocabulary production during the later phase was measured with an abbreviated version of the MacArthur CDI (Toddler version, 16–30 months of age)^13^,^15^ in the discovery cohort (ALSPAC, *N*=6,299, Supplementary Fig. 1b). Note, the Toddler CDI has recently become also known as CDI Words and Sentences^54^. Within the follow-up cohorts, expressive vocabulary was either assessed with the LDS^18^ (GenR *N*=1,812; the Raine study *N*=981) or an adapted short form of the MacArthur CDI (MCDI)^14^,^17^ (Twins Early Development Study, TEDS, *N*=1,727, independent individuals (one twin per pair), N=5,733 twin pairs (not all of them have genotype information available)). Later-phase expressive vocabulary scores measured expressive language only (‘says’) and were either symmetrically distributed or negatively skewed (−1.68<skewness≤0.24; Supplementary Data 1).

In total, three different languages were included in our analyses: English (three samples: ALSPAC; TEDS; Raine), Dutch (one sample: GenR) and Finnish (sensitivity analysis: Northern Finnish Birth Cohort (NFBC) 1966). The cross-cultural comparability of the CDI has been explored, and the measures in many languages, including Dutch and English, show minimal differences in vocabulary production scores in the early years^55^. In addition, the standardization within each sample (see below) would have removed any minor differences between instruments.

Basic study characteristics, details on phenotype acquisition and psychological instruments as well as summary phenotype characteristics (including mean, s.d., kurtosis, skewness and age at measurement) are presented for each cohort and developmental phase in Supplementary Data 1.

For each participating study, ethical approval of the study was obtained by the local research ethics committee, and written informed consent was provided by all parents and legal guardians. Detailed information on sample-specific ethical approval and participant recruitment is provided in Supplementary Note 1.

### Genotyping and imputation

Genotypes within each cohort were obtained using high-density SNP arrays (Supplementary Data 1). Cohort-specific genotyping information including genotyping platform, quality control (QC) for individuals and SNPs, the final sample size, the number of SNPs before and after imputation as well as the imputation procedures are detailed in Supplementary Data 1. Briefly, for individual sample QC, this included filtering according to call rate, heterozygosity and ethnic/other outliers, and for SNP QC (prior to imputation) filtering according to minor allele frequency, call rate and SNPs with deviations from Hardy–Weinberg equilibrium (detailed exclusion criteria are listed in Supplementary Data 1). Genotypes were subsequently imputed to HapMap CEU (phase II and/or III) and/or Wellcome Trust Controls (Supplementary Data 1). For sensitivity analysis, ALSPAC genotypes on chromosome 3 were also locally imputed to 1,000 Genomes (v3.20101123, Supplementary Data 1).

### Single-variant association analysis

Within each cohort, expressive vocabulary scores were adjusted for age, age-squared, sex and the most significant ancestry-informative principal components^56^ and subsequently rank-transformed to normality to facilitate comparison of the data across studies and instruments. The association between SNP and the expressive vocabulary score was assessed within each cohort using linear regression of the rank-transformed expressive vocabulary score against allele dosage, assuming an additive genetic model.

In the discovery cohort, the genome-wide association analysis for each phase was carried out using MACH2QTL^57^ using 2,449,665 imputed or genotyped SNPs. SNPs with a minor allele frequency of <0.01 and SNPs with poor imputation accuracy (MACH *R*^2^≤0.3) were excluded prior to the analysis, and all statistics were subjected to genomic control correction^58^ (Supplementary Data 1). All independent SNPs from the early- and later-phase GWAS below the threshold of *P*<10^−4^ (85 and 50 SNPs, respectively) were selected for subsequent follow-up analysis in additional cohorts. Independent SNPs were identified by linkage disequilibrium-based clumping using PLINK^59^) Proxy SNPs within ±500 kb, linkage disequilibrium *r*^2^>0.3 (Hapmap II CEU, Rel 22) were removed). All analyses within the follow-up samples were carried out *in silico* using MACH2QTL or SNPTEST^60^ software (Supplementary Data 1). For the selected SNPs, estimates from the discovery (genomic-control corrected) and follow-up cohort(s) were combined using fixed-effects inverse-variance meta-analysis (R ‘rmeta’ package), while testing for overall heterogeneity using Cochran’s *Q*-test. Signals below a genome-wide significance threshold of *P*<2.5 × 10^−8^ (accounting for two GWAS analyses) were considered to represent robust evidence for association.

An empirical approach (Bootstrapping with 10,000 replicates) was selected to obtain meaningful genetic effects (basic 95% bootstrap confidence interval) of the reported SNPs in the discovery cohort. For this, we utlilized a linear model of *z*-standardized expressive vocabulary scores against allele dosage, adjusted for age, age-squared, sex and the most significant ancestry-informative principal components. The local departmental server of the School of Social and Community Medicine at the University of Bristol was used for data exchange and storage.

Sensitivity analysis in ALSPAC using locally imputed genotypes on chromosome 3 (based on 1,000 Genomes) was performed as linear regression of the rank-transformed expressive vocabulary score against allele dosage, assuming an additive genetic model, using MACH2QTL (Supplementary Data 1).

### Direct genotyping of reported SNPs

Reported SNPs with a medium imputation accuracy (MACH *R*^2^<0.8) were re-genotyped in the discovery cohort (ALSPAC) to confirm the validity of the observed association signal (rs10734234, MACH *R*^2^=0.76). Genotyping was undertaken by LGC Genomic Ltd ( http://www.lgcgenomics.com/) using a form of competitive allele-specific PCR system (KASPar) for SNP analysis.

### Variance explained

To estimate the variation in expressive vocabulary scores explained by each reported SNP and jointly by all reported SNPs together, we calculated the adjusted regression *R*^2^ values from (i) univariate linear regression of the rank-transformed expressive vocabulary score (see above) against allele dosage and (ii) multivariate linear regression of the rank-transformed expressive vocabulary score (see above) against the allele dosage from all reported SNPs. All analyses were performed using R, SPSS or STATA software.

### Phenotypic characterization of association signals

To investigate whether there is an association between the first single-word utterances at ~12 months of age and the reported SNPs, we conducted an association analysis in the NFBC 1966. The number of spoken words in the NFBC 1966 (word-list free assessment, ‘words’ are undefined) were based on parental response to a questionnaire administered at 12 months of age (Supplementary Data 1). Given the scarcity of categories referring to three or more spoken words, word numbers were dichotomized into ‘1+ words’ (one or more words, 1) versus ‘no words’ (0). The association between early word-production scores and allele dosage of the reported SNPs was studied using logistic regression models, adjusted for sex and the most significant principal components (as exact age at measurement was not available) using SNPTEST.

Pre-school language deficits have been repeatedly associated with later problems in language development, especially reading skills^61^. To assess whether genetic effects affecting expressive language skills early in life also influence language competencies during later development, we investigated the association between reported SNP signals and a series of language-related cognitive measurements in the ALSPAC cohort (Supplementary Table 8). All outcomes were *z*-standardized prior to analysis. The association between the transformed outcome and SNP allele dosage was investigated using linear regression adjusted for sex, the most significant principal components and age (except for age-normalized intelligence quotient scores, Supplementary Table 9).

To assess whether gestational age and maternal education influence the association between the reported signals and early expressive vocabulary scores, we (i) investigated the association between these potential covariates and the SNPs directly and (ii) adjusted the association between genotypes and language measures for potential covariate effects. Gestational age in the relevant cohorts was either estimated from medical records or obtained from midwife and hospital registries at birth (Supplementary Data 1), and measured in completed weeks of gestation. Information on maternal education was obtained from antenatal questionnaire data, and dichotomized into lower (1) and higher (0) maternal education (Supplementary Data 1). The association between gestational age and allele dosage for reported SNPs was investigated with linear regression models and adjusted for sex and the most significant principal components in each cohort. The link between maternal education and these SNPs was studied using logistic regression models adjusted for the most significant principal components in each cohort.

We furthermore created new transformations of expressive vocabulary scores, that is, the reported number of words were in addition to the previously described variables (see above) adjusted for gestational age and maternal education, respectively, before they were rank-transformed. Association analysis for reported SNPs was then carried out as described for discovery, follow-up and combined analysis before. All analyses were carried out using R, SPSS or STATA software.

### GCTA

The proportion of additive phenotypic variation jointly explained by all genome-wide SNPs together (GCTA heritability) was estimated for all cohorts and analyses windows using GCTA^32^. In brief, using a sample of independent individuals, the method is based on the comparison of a matrix of pairwise genomic similarity with a matrix of pairwise phenotypic similarity using a random-effects mixed linear model^32^. Pertinent to this study, GCTA (Supplementary Data 1) was carried out using rank-transformed expressive vocabulary scores (previously adjusted for age, sex and the most significant ancestry-informative principal components in each cohort, see above) and directly genotyped SNPs (ALSPAC, GenR, Raine) or most likely imputed genotypes (TEDS). GCTA estimates from different cohorts were combined using fixed-effects inverse-variance meta-analysis assuming symmetrically distributed s.e., while testing for overall heterogeneity using Cochran’s *Q*-test.

The extent to which the same genes contribute to the observed phenotypic correlation between two variables can be furthermore estimated through genetic correlations^62^. For all cohorts with expressive vocabulary measures at two time points (ALSPAC and GenR), the genetic correlation (*r*_g_) between the rank-transformed scores was estimated using bivariate GCTA analysis^33^ (based on the genetic covariance between two traits).

### Twin analysis

Twin analyses allow the estimation of the relative contributions of genes and environments to individual differences in measured traits. Twin intraclass correlations were calculated^63^, providing an initial indication of the relative contributions of additive genetic (A), shared environmental (C) and non-shared environmental (E) factors. Additive genetic influence, also commonly known as heritability, is estimated as twice the difference between the identical and fraternal twin correlations. The contribution of the shared environment, which makes members of a family similar, is estimated as the difference between the identical twin correlation and heritability. Non-shared environments, that is, environments specific to individuals, are estimated by the difference between the identical twin correlation and 1, because they are the only source of variance making identical twins different. Estimates of the non-shared environment also include measurement error.

Maximum likelihood structural equation model-fitting analyses allow more complex analyses and formal tests of significance^64^. Standard twin model-fitting analyses were conducted using Mx^65^. The model fit is summarized by minus two times the log likelihood (−2LL). Differences in −2LL between models distributes as *χ*^2^, which provides a goodness of fit statistic. A change in *χ*^2^ of 3.84 is significant for a 1 degree of freedom test. Model fit was compared between the full ACE model and the saturated model (where variances are not decomposed into genetic and environmental sources). Reduced models testing CE, AE and E models were compared with the full ACE model and the saturated model. A significant *P* value indicates a significantly worse fit.

Twin analysis was carried out on rank-transformed expressive vocabulary scores at 24 months (adjusted for age, age-squared and sex), which were assessed in 5,733 twin pairs (monozygotic twins *N*=1,969; dizygotic twins (male, female and opposite sex) *N*=3,764) from the TEDS^66^.

The URLs for all utilized web pages are given in Supplementary Note 2.

## Additional information

**How to cite this article**: St Pourcain, B. *et al.* Common variation near *ROBO2* is associated with expressive vocabulary in infancy. *Nat. Commun.* 5:4831 doi: 10.1038/ncomms5831 (2014).

## Supplementary Material

Supplementary Information Supplementary Figures 1-6, Supplementary Tables 1-12, Supplementary Notes 1-3 and Supplementary References

Supplementary Data 1 Basic study characteristics of all cohorts contributing to discovery, follow-up and sensitivity analysis

## Figures and Tables

**Table 1:** Lead association signals for early expressive vocabulary (15–18 months of age).

**SNP**	**E/A**	**Chr**	**Pos** *	**Gene** †	**Discovery (** ***N*** **=6,851)**			**Follow-up (** ***N*** **=2,038)**			**Meta-analysis (** ***N*** **=8,889)**			
					**EAF**	**Beta (s.e.)** ‡	***P*** ‡	**EAF**	**Beta (s.e.)**	***P***	**EAF**	**Beta (s.e.)**	***P***	***P***_**het**_
rs7642482	G/A	3p12.3	77,800,446	* ROBO2 *	0.18	−0.11 (0.022)	9.5 × 10^−7^	0.19	−0.12 (0.040)	4.4 × 10^−3^	0.19	−0.11 (0.019)	1.3 × 10^−8^	0.90
rs10734234	T/C	11p15.2	15,422,436	* INSC *	0.90	−0.14 (0.032)	1.1 × 10^−5^	0.90	−0.17 (0.059)	4.5 × 10^−3^	0.90	−0.15 (0.028)	1.9 × 10^−7^	0.72
rs11176749	T/A	12q15	66,139,051	* CAND1 *	0.11	−0.12 (0.027)	2.1 × 10^−5^	0.11	−0.13 (0.050)	1.0 × 10^−2^	0.11	−0.12 (0.024)	7.2 × 10^−7^	0.83
rs1654584	G/T	19p13.3	3,921,683	* DAPK3 *	0.23	−0.081 (0.020)	6.2 × 10^−5^	0.23	−0.13 (0.038)	9.2 × 10^−4^	0.23	−0.091 (0.018)	3.4 × 10^−7^	0.30

A, alternative allele; ALSPAC, Avon Longitudinal Study of Parents and Children; CDI, Communicative Development Inventory; Chr, chromosome; E, effect allele; EAF, effect allele frequency; Pos, position; *P*_het,_ heterogeneity *P-*value.

Genome-wide screen of rank-transformed expressive vocabulary scores between 15–18 months of age in children of European ancestry. Discovery analysis was conducted in ALSPAC (Abbreviated Infant CDI^13^) and independent signals were followed-up in GenR (N-CDI-2A^14^,^16^). Combined results are from inverse-variance fixed-effect meta-analysis. Beta coefficients represent the change in the rank-transformed score (adjusted for sex, age, age-squared and the most significant principal components in each cohort) per effect allele from weighted linear regression of the score on allele dosage (MACH2QTL). The imputation accuracy (Supplementary Table 3) for rs7642482, rs11176749 and rs1654584 was high (MACH *R*^2^≥0.95), and for rs10734234 moderate (MACH *R*^2^=0.75–0.76). Thus, the signal at rs10734234 in the discovery cohort was confirmed by direct genotyping (Supplementary Table 4).

*P*_het_—heterogeneity *P-*value based on Cochran’s *Q*-test.

^*^hg18.

^†^Nearest known gene within ±500 kb.

^‡^Genomic-control corrected.

**Table 2:** Heritability of expressive vocabulary (15–30 months).

**Sample**	**Age (m)**	**Measure**	***h*** ^**2**^ **(s.e.)** *	**LRT (df)**	***P***	***N*** †
*GCTA: early expressive vocabulary (15–18 months)*						
ALSPAC	15	Infant CDI	0.13 (0.05)	5.66 (1)	0.009	6,194
GenR	18	N-CDI-2A	0.19 (0.17)	1.23 (1)	0.10	1,828
Total‡			0.13 (0.05)			8,022
						
*GCTA: later expressive vocabulary (24–30 months)*						
ALSPAC	24	Toddler CDI	0.17 (0.06)	8.09 (1)	0.002	5,739
Raine	24	LDS	<0.01 (0.34)	<0.01 (1)	0.50	866
TEDS	24	MCDI	<0.01 (0.15)	<0.01 (1)	0.50	1,720
GenR	30	LDS	0.11 (0.19)	0.33 (1)	0.30	1,641
Total‡			0.14 (0.05)			9,966

**Sample**	**Age (m)**	**Measure**	***a*** ^**2**^ **(s.e.)** §			***N*** ||
*Twin analysis: later expressive vocabulary (24 months)*						
TEDS	24	MCDI	0.20 (0.008)			5,733

Abbreviations: ALSPAC, Avon Longitudinal Study of Parents and Children; GCTA, genome-wide complex trait analysis; m, months; TEDS, Twins Early Development Study.

Expressive vocabulary was captured with different forms of the MacArthur Communicative Development Inventories (CDI: infant CDI, toddler CDI, N-CDI-2A and MCDI)^13^,^14^,^15^,^16^,^17^ and the Language development Survey (LDS)^18^ (Supplementary Data 1).

^*^GCTA heritability based on rank-transformed expressive vocabulary scores adjusted for age, age-squared, sex and the most significant ancestry-informative principal components in each cohort.

^†^Sample number after exclusion of individuals with a relatedness of ≥2.5%.

^‡^Estimates were combined using fixed-effects inverse-variance meta-analysis (heterogeneity *P* value based on Cochran’s *Q*-test based *P*_het_≥0.72).

^§^Additive genetic influence for rank-transformed expressive vocabulary scores adjusted for age, age-squared and sex, based on an ACE model (Supplementary Tables 10 and 11).

^||^Number of twin pairs.
