# Supplementary material for: Common variation near ROBO2 is associated with expressive vocabulary in infancy
Source: Nat Commun. 2014 Sep 16;5:4831. doi: 10.1038/ncomms5831 (PMC4175587; doi:10.1038/ncomms5831)
Supplement: Supplementary Information — Supplementary Figures 1-6, Supplementary Tables 1-12, Supplementary Notes 1-3 and Supplementary References [file ncomms5831-s1.pdf]

SUPPLEMENTARY FIGURES

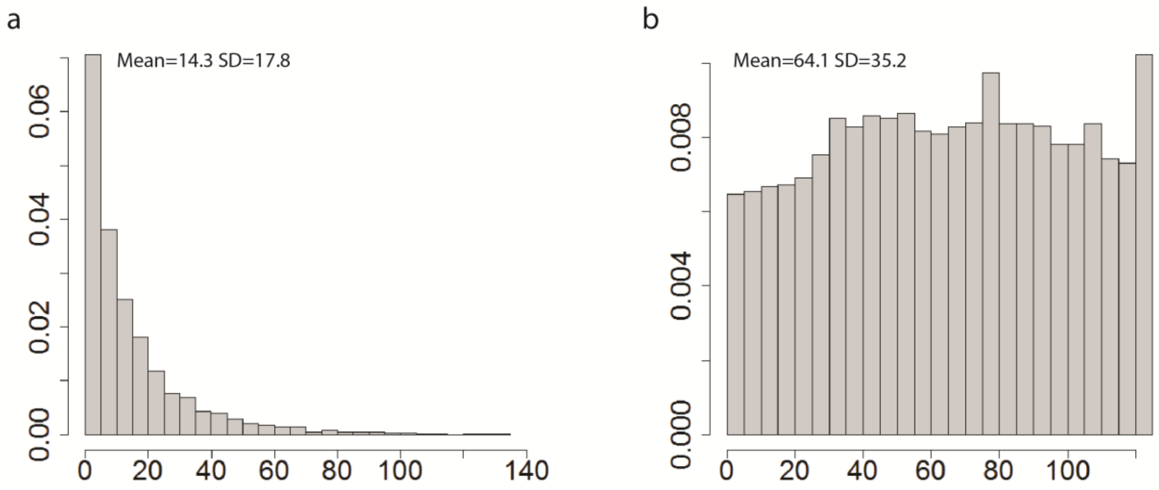

Supplementary Figure 1. Phenotype distribution of expressive vocabulary in the discovery cohort Measures were ascertained within ALSPAC at 15 months (a) and 24 months of age (b). Expressive vocabulary during the early phase was captured by an abbreviated version of the MacArthur CDI (Infant Version[1], 8-16 months of age), and vocabulary production during the later phase was measured with an abbreviated version of the MacArthur CDI (Toddler version, 16 to 30 months of age)[1,2]. Detailed phenotype descriptions are given in **Supplementary Data 1**.

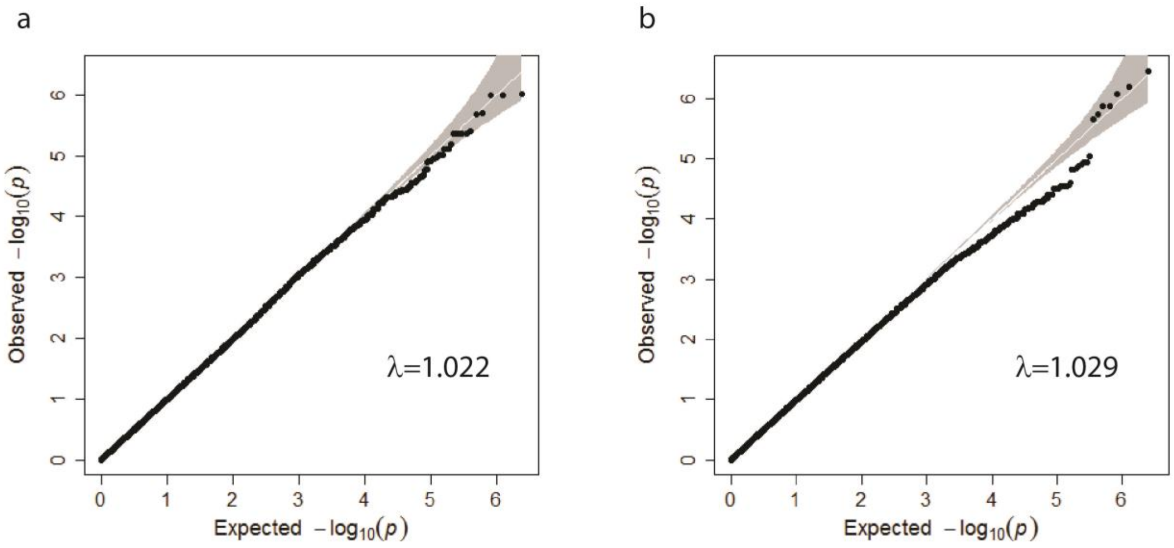

Supplementary Figure 2. Quantile-quantile plots of genome-wide signals in the discovery cohort

Genome-wide analysis (2,449,665 SNPs) within ALSPAC was carried out for an early (a, 15-18 months, N=6,851) and a later (b, 24-30 months, N=6,299) phase of language acquisition. Black circles depict the observed association signals (*p*-values), the white diagonal line represents the distribution of signals under the null hypothesis and the shaded area corresponds to the 95% confidence interval.

λ – Genomic-control factor

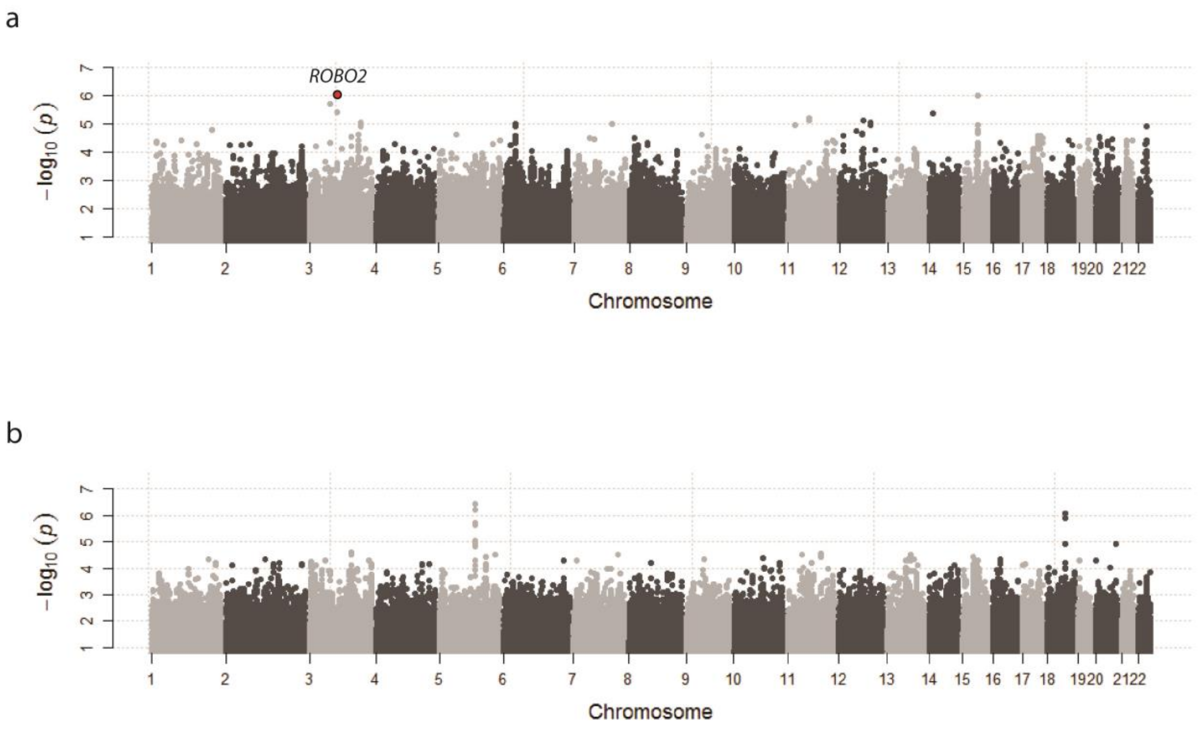

Supplementary Figure 3. Manhattan plots of genome-wide signals in the discovery cohort Genome-wide analysis (2,449,665 SNPs) within ALSPAC was carried out for an early (a, 15-18 months, N=6,851) and a later (b, 24-30 months, N=6,299) phase of language acquisition. $-\log_{10}$ *p*-values are plotted against their genomic position (hg18). Association signals with genome-significance in the meta-analysis of discovery and follow-up cohorts are shown in red.

a

Hapmap II, hg18

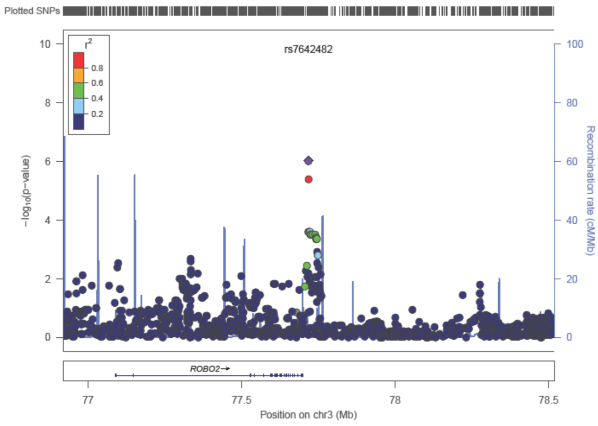

b

1000 Genomes, hg19

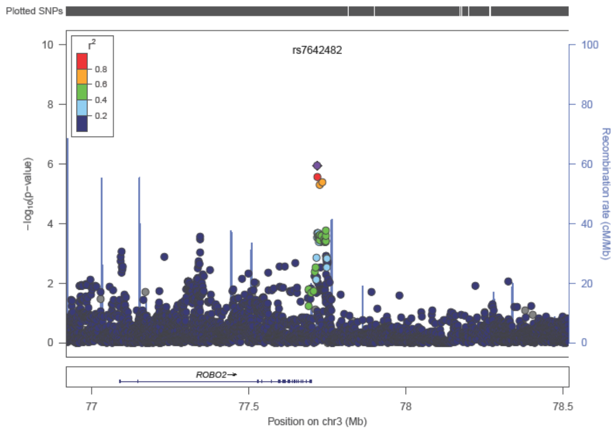

Supplementary Figure 4: Regional association plots at 3p12.3 for early expressive vocabulary using different imputation templates

Association analysis was conducted in ALSPAC using a) Hapmap II imputed genotypes (hg18) and b) 1000 Genomes imputed genotypes (v3.20101123, hg19, **Supplementary Data 1**). SNPs are plotted with their -$\log_{10}$ *p*-value as a function of the genomic position. *P*-values were generated from weighted linear regression of the rank-transformed vocabulary score (15-18 months of age) on allele dosage. The local linkage disequilibrium (LD) structure near the associated region is reflected by recombination rates estimated from (a) Hapmap II or (b) 1000 Genomes. SNPs are coloured on the basis of their correlation with the lead signal (based on pair-wise r^2 values).

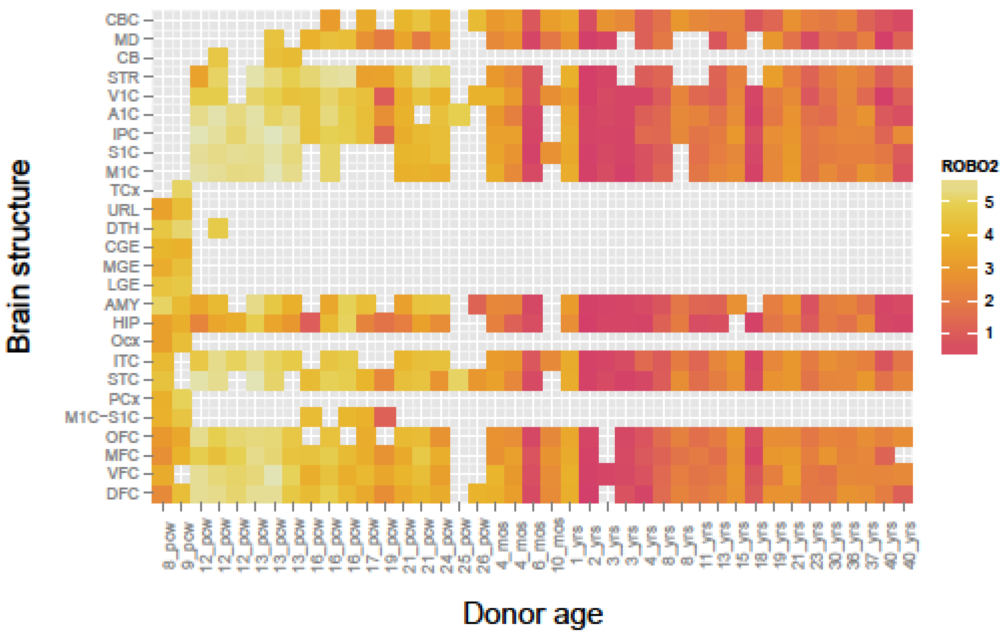

Supplementary Figure 5. Developmental expression profile of *ROBO2* in brain

The *ROBO2* mRNA expression profile (Brainspan, http://www.brainspan.org/) is reported as log2 RPKM (reads per kilobase per million) according to donor tissue and donor age (8 weeks of gestation – 41 years). DFC - dorsolateral prefrontal cortex, VFC - ventrolateral prefrontal cortex, MFC - anterior (rostral) cingulate (medial prefrontal) cortex, OFC - orbital frontal cortex, M1C-S1C - primary motor-sensory cortex (samples), PCx - parietal neocortex, STC - posterior (caudal) superior temporal cortex, ITC - inferolateral temporal cortex, Ocx - occipital neocortex, HIP - hippocampus (hippocampal formation), AMY - amygdaloid complex, LGE - lateral ganglionic eminence, MGE - medial ganglionic eminence, CGE- caudal ganglionic eminence, DTH - dorsal thalamus, URL - upper (rostral) rhombic lip, TCx - temporal neocortex, Ocx - occipital neocortex, M1C - primary motor cortex, S1C - primary somatosensory cortex, IPC - posteroventral (inferior) parietal cortex, A1C - primary auditory cortex, V1C - primary visual cortex, STR – striatum, CB – Cerebellum, MD - mediodorsal nucleus of thalamus, CBC - cerebellar cortex, pcw – week gestation, mos – months, yrs - years

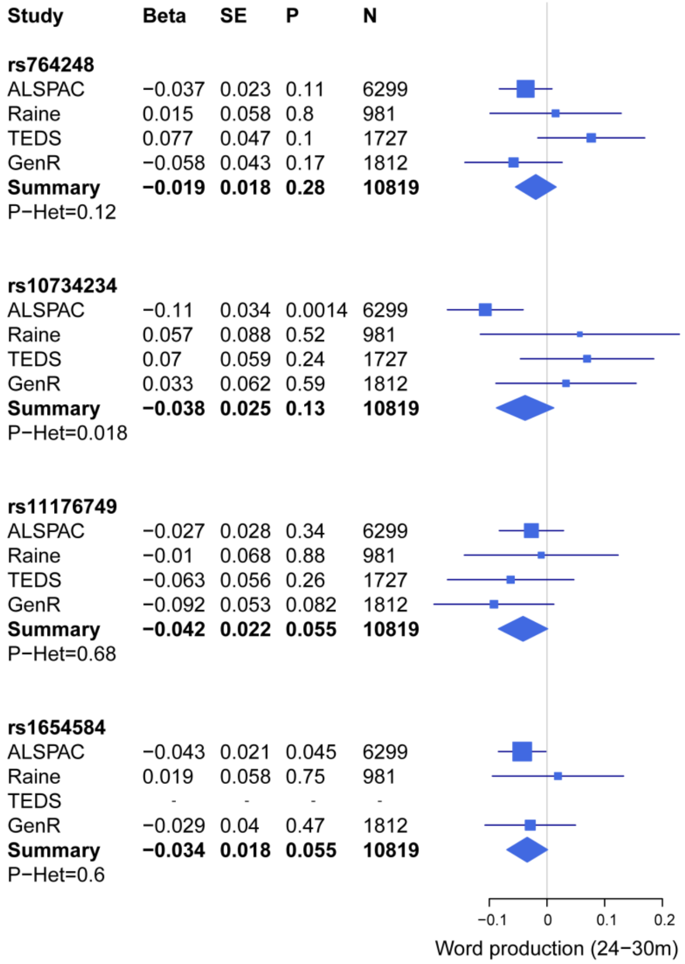

Supplementary Figure 6. Association between lead association signals for early expressive vocabulary and later expressive vocabulary scores between 24 and 30 months

Forest plots include results from ALSPAC (Genomic-control corrected), Raine, TEDS and GenR and an inverse-variance fixed effect meta-analysis of all cohorts. Beta coefficients represent the change in rank-transformed expressive vocabulary score (adjusted for sex, age, age squared and the most significant principal components in each cohort) per effect allele from weighted linear regression of the score on allele dosage (MACH2QTL/SNPTEST). Effects are given with respect to the following effect alleles: rs7642482 (G), rs10734234 (T), rs11176749 (T) and rs1654584 (G).

SUPPLEMENTARY TABLES

Supplementary Table 1. Association signals for early expressive vocabulary (15-18 months, $p \leq 10^{-4}$)

				Discovery(N=6851)			Meta-analysis (N=8889)			
**SNP**	**E/A**	**Chr**	**Pos[a]**	**EAF**	**Beta(SE)[b]**	***p*[b]**	**EAF**	**Beta(SE)**	***p***	**Dir**
rs11804375	T/C	1	16,001,561	0.11	0.12(0.028)	4.2×10^{-5}	0.11	0.099(0.025)	6.0×10^{-5}	++
rs11208754	A/G	1	40,948,896	0.89	0.11(0.028)	5.6×10^{-5}	0.88	0.09(0.024)	2.1×10^{-4}	++
rs11810174	T/C	1	98,907,589	0.94	0.16(0.038)	3.9×10^{-5}	0.94	0.1(0.034)	3.2×10^{-3}	+-
rs1234318	A/G	1	171,364,058	0.65	-0.082(0.02)	5.0×10^{-5}	0.65	-0.056(0.018)	1.7×10^{-3}	-+
rs1498029	A/C	1	217,150,795	0.98	0.29(0.067)	1.7×10^{-5}	0.98	0.25(0.06)	3.6×10^{-5}	++
rs4669286	A/G	2	8,612,388	0.10	-0.16(0.039)	5.5×10^{-5}	0.09	-0.12(0.034)	7.0×10^{-4}	-+
rs4952624	A/C	2	40,506,606	0.97	0.22(0.055)	6.0×10^{-5}	0.97	0.17(0.048)	5.4×10^{-4}	+-
rs13420384	A/G	2	59,642,081	0.08	-0.14(0.033)	5.2×10^{-5}	0.08	-0.12(0.029)	3.8×10^{-5}	--
rs16826639	C/G	2	230,704,789	0.74	0.077(0.019)	6.1×10^{-5}	0.74	0.049(0.017)	3.9×10^{-3}	+-
rs4684234	A/G	3	14,596,299	0.37	0.081(0.02)	6.2×10^{-5}	0.38	0.073(0.018)	4.3×10^{-5}	++
rs12487696	A/G	3	59,538,614	0.78	-0.1(0.021)	2.0×10^{-6}	0.79	-0.078(0.019)	2.8×10^{-5}	-0
rs17061008	A/G	3	59,618,671	0.54	0.074(0.018)	4.8×10^{-5}	0.53	0.058(0.016)	2.9×10^{-4}	++
**rs7642482**	**A/G**	**3**	**77,800,446**	**0.82**	**0.11(0.022)**	$\mathbf{9.5\times10^{-7}}$	**0.81**	**0.11(0.019)**	$\mathbf{1.3\times10^{-8}}$	**++**
rs1874655	A/C	3	99,552,155	0.75	0.08(0.02)	7.6×10^{-5}	0.75	0.056(0.018)	1.5×10^{-3}	+-
rs4234700	A/G	3	130,989,172	0.90	0.12(0.028)	3.1×10^{-5}	0.90	0.08(0.025)	1.5×10^{-3}	+-
rs1006742	A/G	3	152,108,938	0.84	-0.16(0.041)	8.4×10^{-5}	0.84	-0.084(0.036)	1.9×10^{-2}	-+
rs9857706	A/G	3	152,129,866	0.29	-0.081(0.019)	2.5×10^{-5}	0.29	-0.061(0.017)	2.8×10^{-4}	-+
rs13073941	T/C	3	160,680,164	0.27	-0.085(0.019)	9.6×10^{-6}	0.27	-0.062(0.017)	2.3×10^{-4}	-+
rs792354	T/C	3	174,456,839	0.34	-0.072(0.018)	7.6×10^{-5}	0.34	-0.059(0.016)	2.0×10^{-4}	--
rs2537921	T/C	4	18,588,912	0.27	-0.075(0.019)	9.4×10^{-5}	0.27	-0.071(0.017)	2.9×10^{-5}	--
rs6814920	A/G	4	58,173,417	0.23	0.082(0.02)	5.0×10^{-5}	0.23	0.056(0.018)	1.6×10^{-3}	+-
rs342435	A/G	4	88,262,230	0.41	0.068(0.017)	7.6×10^{-5}	0.41	0.06(0.015)	8.1×10^{-5}	++
rs7694786	A/C	4	187,916,334	0.67	-0.076(0.019)	7.6×10^{-5}	0.67	-0.054(0.017)	1.3×10^{-3}	-+
rs2652492	T/C	5	5,967,817	0.37	0.075(0.019)	9.4×10^{-5}	0.37	0.06(0.017)	4.6×10^{-4}	++
rs13164951	T/G	5	50,015,814	0.9	-0.13(0.03)	2.4×10^{-5}	0.9	-0.11(0.027)	2.2×10^{-5}	--
rs16750	A/G	5	159,062,555	0.87	0.1(0.025)	5.4×10^{-5}	0.87	0.085(0.022)	1.4×10^{-4}	++
rs6894268	A/G	5	178,965,094	0.32	-0.075(0.018)	3.8×10^{-5}	0.32	-0.048(0.016)	2.9×10^{-3}	-+
rs4715888	A/G	6	14,462,126	0.31	0.073(0.018)	6.0×10^{-5}	0.31	0.051(0.016)	1.7×10^{-3}	+-
rs3799344	T/C	6	25,894,972	0.44	-0.076(0.017)	9.8×10^{-6}	0.44	-0.048(0.015)	1.6×10^{-3}	-+
rs314214	A/G	6	69,935,117	0.82	0.087(0.022)	9.2×10^{-5}	0.82	0.079(0.02)	5.7×10^{-5}	++
rs1340080	A/G	6	164,754,183	0.13	-0.1(0.026)	8.9×10^{-5}	0.13	-0.082(0.023)	3.7×10^{-4}	--
rs4720231	A/C	7	36,955,623	0.27	-0.08(0.019)	3.1×10^{-5}	0.28	-0.062(0.017)	2.0×10^{-4}	--
rs921908	T/C	7	50,212,082	0.76	0.092(0.022)	3.5×10^{-5}	0.76	0.047(0.019)	1.6×10^{-2}	+-
rs7005662	T/C	8	6,464,034	0.29	-0.08(0.019)	3.1×10^{-5}	0.29	-0.061(0.017)	2.7×10^{-4}	-+
rs2201476	A/G	8	15,302,254	0.39	-0.073(0.018)	6.0×10^{-5}	0.39	-0.058(0.016)	2.4×10^{-4}	--
rs7015219	A/G	8	38,939,594	0.55	-0.07(0.017)	4.6×10^{-5}	0.54	-0.059(0.015)	8.3×10^{-5}	--
rs1897446	A/C	8	130,530,423	0.35	-0.071(0.018)	9.5×10^{-5}	0.34	-0.047(0.016)	3.1×10^{-3}	-+
rs7861219	T/C	9	28,199,655	0.17	0.098(0.023)	2.5×10^{-5}	0.17	0.078(0.02)	1.2×10^{-4}	++
rs7851060	A/T	9	75,612,036	0.82	-0.093(0.023)	6.3×10^{-5}	0.82	-0.076(0.02)	1.9×10^{-4}	--
rs10820378	T/C	9	98,132,570	0.84	-0.092(0.023)	7.6×10^{-5}	0.84	-0.07(0.02)	5.6×10^{-4}	-0
rs10759964	A/G	9	120,090,898	0.36	0.071(0.018)	9.5×10^{-5}	0.36	0.054(0.016)	7.1×10^{-4}	+-
rs12359510	T/C	10	9,446,646	0.29	-0.076(0.019)	7.6×10^{-5}	0.29	-0.051(0.017)	2.6×10^{-3}	-+
**rs10734234**	**T/C**	**11**	**15,422,436**	**0.9**	**-0.14(0.032)**	$\mathbf{1.1\times10^{-5}}$	**0.9**	**-0.15(0.028)**	$\mathbf{1.9\times10^{-7}}$	**--**
rs198750	T/C	11	61,144,666	0.11	-0.12(0.027)	6.6×10^{-6}	0.11	-0.1(0.024)	2.7×10^{-5}	--

Supplementary Table 1 (cont'd). Association signals for early expressive vocabulary (15-18 months, $p \leq 10^{-4}$)

				Discovery(N=6851)			Meta-analysis (N=8889)			
SNP	E/A	Chr	Pos[a]	EAF	Beta(SE)[b]	p^b	EAF	Beta(SE)	p	Dir
rs2726888	T/G	11	108,142,998	0.13	-0.099(0.025)	9.0×10^{-5}	0.13	-0.086(0.022)	1.2×10^{-4}	--
rs7113211	A/G	11	112,147,842	0.5	-0.078(0.019)	4.9×10^{-5}	0.5	-0.073(0.017)	8.7×10^{-6}	--
rs11221096	T/C	11	127,254,104	0.08	-0.19(0.046)	3.9×10^{-5}	0.08	-0.14(0.04)	5.3×10^{-4}	-+
rs318961	T/C	11	130,869,705	0.78	0.083(0.021)	9.2×10^{-5}	0.78	0.061(0.019)	1.0×10^{-3}	+-
rs893951	T/G	11	133,763,626	0.32	-0.074(0.018)	4.8×10^{-5}	0.33	-0.054(0.016)	7.3×10^{-4}	-+
rs10845602	A/G	12	12,716,504	0.59	0.072(0.017)	2.8×10^{-5}	0.59	0.064(0.015)	2.1×10^{-5}	++
rs11608516	T/C	12	47,074,366	0.05	0.23(0.054)	1.8×10^{-5}	0.05	0.2(0.046)	1.9×10^{-5}	++
rs10878615	C/G	12	66,118,859	0.16	-0.091(0.023)	9.1×10^{-5}	0.16	-0.078(0.02)	1.3×10^{-4}	--
**rs11176749**	**A/T**	**12**	**66,139,051**	**0.89**	**0.12(0.027)**	$\mathbf{2.1 \times 10^{-5}}$	**0.89**	**0.12(0.024)**	$\mathbf{7.2 \times 10^{-7}}$	**++**
rs7306190	A/G	12	69,455,688	0.83	0.1(0.023)	7.7×10^{-6}	0.83	0.089(0.021)	1.6×10^{-5}	++
rs11106179	A/G	12	90,420,543	0.20	-0.094(0.021)	9.5×10^{-6}	0.20	-0.087(0.019)	3.4×10^{-6}	--
rs2453165	T/C	12	103,737,294	0.97	-0.25(0.065)	8.6×10^{-5}	0.97	-0.22(0.057)	1.0×10^{-4}	--
rs4772487	T/G	13	85,903,906	0.34	-0.072(0.018)	7.6×10^{-5}	0.34	-0.05(0.016)	1.8×10^{-3}	-+
rs17096641	A/C	14	29,926,756	0.92	-0.14(0.031)	4.3×10^{-6}	0.92	-0.12(0.028)	1.9×10^{-5}	--
rs11632744	T/C	15	59,701,327	0.72	0.087(0.022)	9.2×10^{-5}	0.72	0.055(0.019)	4.1×10^{-3}	+-
rs12148373	A/G	15	63,321,342	0.52	0.084(0.017)	1.0×10^{-6}	0.52	0.065(0.015)	2.1×10^{-5}	+-
rs8025474	T/C	15	66,173,577	0.31	0.08(0.018)	1.1×10^{-5}	0.3	0.059(0.016)	2.8×10^{-4}	+-
rs12920537	A/G	16	20,329,057	0.72	0.078(0.019)	4.9×10^{-5}	0.72	0.046(0.017)	6.2×10^{-3}	+-
rs187680	A/C	16	50,981,658	0.6	0.068(0.017)	7.6×10^{-5}	0.6	0.064(0.015)	2.4×10^{-5}	++
rs12445038	T/C	16	56,817,301	0.04	-0.21(0.053)	9.6×10^{-5}	0.04	-0.13(0.044)	3.9×10^{-3}	-+
rs7209108	T/C	17	44,218,897	0.26	0.076(0.019)	7.6×10^{-5}	0.26	0.069(0.017)	5.4×10^{-5}	++
rs2465411	A/G	17	58,063,009	0.41	0.072(0.017)	2.8×10^{-5}	0.4	0.053(0.015)	4.2×10^{-4}	+-
rs6501538	T/C	17	67,885,084	0.5	0.072(0.017)	2.8×10^{-5}	0.5	0.057(0.015)	1.7×10^{-4}	++
rs11657164	T/C	17	67,885,977	0.83	0.092(0.023)	7.6×10^{-5}	0.83	0.079(0.02)	1.2×10^{-4}	++
rs9907431	A/G	17	74,071,124	0.34	-0.084(0.02)	3.3×10^{-5}	0.34	-0.054(0.018)	2.5×10^{-3}	-+
rs4395181	T/C	18	62,952,025	0.38	0.075(0.018)	3.8×10^{-5}	0.38	0.065(0.016)	4.6×10^{-5}	++
rs10514093	A/G	18	69,667,638	0.98	0.37(0.093)	5.8×10^{-5}	0.98	0.28(0.08)	5.8×10^{-4}	+-
**rs1654584**	**T/G**	**19**	**3,921,683**	**0.77**	**0.081(0.02)**	$\mathbf{6.2 \times 10^{-5}}$	**0.77**	**0.091(0.018)**	$\mathbf{3.4 \times 10^{-7}}$	**++**
rs3865452	T/C	19	45,902,896	0.5	0.07(0.017)	4.6×10^{-5}	0.5	0.052(0.015)	6.0×10^{-4}	+-
rs12463085	A/G	19	51,161,251	0.69	-0.079(0.019)	3.9×10^{-5}	0.69	-0.053(0.017)	1.5×10^{-3}	-+
rs11697413	A/G	20	1,213,152	0.06	-0.14(0.036)	9.5×10^{-5}	0.06	-0.12(0.032)	1.7×10^{-4}	--
rs2273189	C/G	20	8,613,751	0.95	0.2(0.048)	2.8×10^{-5}	0.95	0.15(0.042)	4.5×10^{-4}	+-
rs6032829	T/C	20	10,178,291	0.16	0.098(0.024)	5.4×10^{-5}	0.16	0.07(0.021)	9.7×10^{-4}	+-
rs11699690	A/G	20	33,735,188	0.09	0.13(0.031)	4.4×10^{-5}	0.09	0.095(0.028)	5.4×10^{-4}	+-
rs11700299	T/C	20	34,012,279	0.11	0.12(0.028)	4.2×10^{-5}	0.11	0.077(0.025)	1.9×10^{-3}	+-
rs6066696	A/T	20	46,386,093	0.33	-0.072(0.018)	7.6×10^{-5}	0.33	-0.063(0.016)	8.4×10^{-5}	--
rs802952	T/C	20	47,519,676	0.56	-0.071(0.017)	3.6×10^{-5}	0.56	-0.046(0.015)	2.3×10^{-3}	-+
rs8130870	T/C	21	23,803,965	0.75	0.083(0.02)	4.0×10^{-5}	0.75	0.074(0.018)	2.6×10^{-5}	++
rs4818184	A/G	21	41,124,301	0.13	-0.1(0.025)	3.9×10^{-5}	0.13	-0.1(0.022)	7.0×10^{-6}	--
rs132503	T/C	22	37,616,345	0.8	0.09(0.022)	5.2×10^{-5}	0.8	0.068(0.02)	5.2×10^{-4}	+-
rs713628	C/G	22	42,012,214	0.7	0.084(0.019)	1.2×10^{-5}	0.71	0.061(0.017)	2.8×10^{-4}	+-

Genome-wide screen of expressive vocabulary scores between 15-18 months of age. Discovery analysis was conducted in ALSPAC and independent signals ($p \leq 1 \times 10^{-4}$) were followed up in GenR (N=2038; **Supplementary Data 1**). Combined results are from inverse-variance fixed effect meta-analysis. Beta coefficients represent the change in rank-transformed score (adjusted for sex, age, age squared and the most significant principal components in each cohort) per effect allele from weighted linear regression of the score on allele dosage (MACH2QTL). Lead signals are indicated in bold. E – Effect allele, A – Alternative allele, Chr – Chromosome, Pos – Position, EAF – Effect allele frequency, Dir – Direction of the genetic effect; a - hg18, b - Genomic-control corrected

Supplementary Table 2. Association signals for later expressive vocabulary (24-30 months, $p \leq 10^{-4}$)

				Discovery(N=6299)			Meta-analysis(N=10819)			
**SNP**	**E/A**	**Chr**	**Pos**[a]	**EAF**	**Beta(SE)**[b]	***p***[b]	**EAF**	**Beta(SE)**	***p***	**Dir**
rs12410765	A/T	1	206,254,554	0.87	-0.12(0.03)	$4.6x10^{-5}$	0.87	-0.086(0.025)	$7.7x10^{-4}$	-+-? [c]
rs16850132	A/G	1	227,902,789	0.7	0.077(0.019)	$6.5x10^{-5}$	0.7	0.052(0.015)	$4.3x10^{-4}$	+++-
rs449993	A/G	2	14,095,240	0.96	0.23(0.059)	$8.0x10^{-5}$	0.96	0.16(0.044)	$3.8x10^{-4}$	+-++
rs10496465	A/G	2	114,974,533	0.85	0.099(0.024)	$4.8x10^{-5}$	0.85	0.054(0.019)	$4.6x10^{-3}$	+-+-
rs1561444	T/G	2	139,745,456	0.25	-0.085(0.021)	$6.6x10^{-5}$	0.25	-0.029(0.016)	$7.0x10^{-2}$	++-+
rs17207382	A/G	2	159,666,010	0.63	0.073(0.018)	$6.4x10^{-5}$	0.63	0.041(0.014)	$3.1x10^{-3}$	+++-
rs16825679	T/C	2	229,661,770	0.16	0.097(0.024)	$6.8x10^{-5}$	0.17	0.060(0.018)	$1.1x10^{-3}$	++++
rs6791159	C/G	3	2,905,924	0.11	-0.14(0.034)	$5.5x10^{-5}$	0.11	-0.11(0.027)	$1.2x10^{-4}$	---? [c]
rs7629002	A/C	3	14,580,991	0.31	-0.076(0.019)	$8.0x10^{-5}$	0.31	-0.038(0.015)	$9.9x10^{-3}$	++--
rs9837325	A/C	3	130,798,521	0.2	-0.088(0.022)	$8.0x10^{-5}$	0.2	-0.040(0.017)	$1.9x10^{-2}$	++--
rs16889	A/C	3	131,196,174	0.49	-0.077(0.018)	$2.5x10^{-5}$	0.48	-0.041(0.014)	$3.1x10^{-3}$	+---
rs1512077	A/G	3	147,575,442	0.3	0.075(0.019)	$1.0x10^{-4}$	0.3	0.051(0.015)	$6.4x10^{-4}$	-+++
rs10937169	T/C	3	185,613,725	0.42	-0.074(0.018)	$5.1x10^{-5}$	0.42	-0.045(0.014)	$1.2x10^{-3}$	+---
rs7650510	A/G	3	191,845,810	0.76	0.087(0.022)	$9.7x10^{-5}$	0.76	0.051(0.017)	$2.1x10^{-3}$	-+++
rs4305462	A/T	3	192,013,125	0.19	-0.1(0.025)	$6.8x10^{-5}$	0.18	-0.052(0.019)	$6.7x10^{-3}$	++--
rs1395821	T/C	4	148,267,000	0.82	0.093(0.023)	$6.7x10^{-5}$	0.82	0.070(0.018)	$8.9x10^{-5}$	+-++
rs9312489	A/C	4	171,874,489	0.23	0.085(0.021)	$6.6x10^{-5}$	0.23	0.049(0.016)	$2.5x10^{-3}$	+-+-
rs11742977	C/G	5	110,624,391	0.59	0.093(0.018)	$3.5x10^{-7}$	0.59	0.065(0.014)	$3.5x10^{-6}$	++++
rs13182561	A/G	5	140,624,668	0.80	0.096(0.023)	$3.9x10^{-5}$	0.8	0.055(0.018)	$1.8x10^{-3}$	--++
rs9371371	A/G	6	155,666,796	0.4	-0.078(0.019)	$5.2x10^{-5}$	0.41	-0.033(0.014)	$2.0x10^{-2}$	++-+
rs10155941	T/C	7	134,073,272	0.54	-0.076(0.018)	$3.2x10^{-5}$	0.54	-0.046(0.014)	$9.8x10^{-4}$	-+-+
rs6981525	A/G	8	57,772,551	0.46	-0.077(0.019)	$6.5x10^{-5}$	0.47	-0.036(0.015)	$1.4x10^{-2}$	+--+
rs2182746	T/C	9	37,521,193	0.05	0.18(0.044)	$4.5x10^{-5}$	0.05	0.11(0.033)	$1.1x10^{-3}$	+++-
rs1863898	A/G	10	79,449,156	0.48	0.075(0.018)	$4.0x10^{-5}$	0.48	0.054(0.014)	$1.0x10^{-4}$	++++
rs10748710	A/G	10	83,084,355	0.29	0.083(0.021)	$9.8x10^{-5}$	0.29	0.058(0.016)	$2.9x10^{-4}$	++++
rs2421140	A/T	10	124,777,360	0.03	-0.23(0.057)	$6.0x10^{-5}$	0.03	-0.088(0.042)	$3.8x10^{-2}$	++--
rs7929580	A/G	11	36,415,193	0.88	-0.11(0.027)	$3.2x10^{-5}$	0.88	-0.081(0.021)	$1.1x10^{-4}$	---+
rs2510894	T/G	11	74,739,826	0.47	0.073(0.018)	$6.4x10^{-5}$	0.47	0.03(0.014)	$3.2x10^{-2}$	+-+-
rs12796629	T/G	11	98,205,037	0.01	-0.38(0.091)	$2.7x10^{-5}$	0.01	-0.25(0.07)	$3.5x10^{-4}$	---+
rs17532216	T/G	13	66,968,886	0.9	-0.12(0.03)	$5.3x10^{-5}$	0.9	-0.065(0.023)	$5.1x10^{-3}$	-+-+
rs9600102	T/C	13	72,709,016	0.06	0.16(0.04)	$5.2x10^{-5}$	0.06	0.086(0.03)	$4.0x10^{-3}$	-+++
rs924895	T/C	13	75,804,599	0.55	-0.076(0.018)	$3.2x10^{-5}$	0.56	-0.041(0.014)	$3.4x10^{-3}$	-+-+
rs7320524	T/G	13	87,184,488	0.19	0.099(0.024)	$4.8x10^{-5}$	0.18	0.079(0.019)	$2.9x10^{-5}$	-+++
rs11617458	A/G	13	97,665,446	0.74	0.08(0.02)	$8.0x10^{-5}$	0.74	0.04(0.016)	$1.1x10^{-2}$	--++
rs8020079	A/G	14	89,980,698	0.95	-0.17(0.043)	$8.0x10^{-5}$	0.95	-0.13(0.036)	$2.9x10^{-4}$	+--? [c]
rs8034690	A/G	15	51,350,929	0.83	0.1(0.025)	$6.8x10^{-5}$	0.83	0.042(0.019)	$2.7x10^{-2}$	--+-
rs1280396	A/G	15	55,519,020	0.19	0.094(0.023)	$5.6x10^{-5}$	0.19	0.044(0.018)	$1.3x10^{-2}$	--+-
rs16946198	A/C	15	61,055,589	0.92	-0.14(0.035)	$7.1x10^{-5}$	0.92	-0.089(0.026)	$5.8x10^{-4}$	+--+
rs8031639	A/C	15	64,763,977	0.57	-0.074(0.018)	$5.1x10^{-5}$	0.57	-0.051(0.014)	$2.8x10^{-4}$	----
rs12600092	A/G	16	20,057,104	0.77	0.087(0.021)	$4.4x10^{-5}$	0.78	0.067(0.017)	$4.9x10^{-5}$	++++
rs2447098	A/C	17	2,224,470	0.52	0.08(0.02)	$8.0x10^{-5}$	0.51	0.059(0.015)	$1.1x10^{-4}$	-+++
rs3816577	T/C	17	9,289,321	0.23	0.085(0.021)	$6.6x10^{-5}$	0.23	0.036(0.016)	$2.8x10^{-2}$	-++-
rs4594325	A/G	18	2,451,449	0.29	-0.079(0.02)	$9.9x10^{-5}$	0.29	-0.062(0.015)	$5.1x10^{-5}$	+---
rs11151979	T/C	18	52,558,537	0.23	0.11(0.022)	$8.3x10^{-7}$	0.22	0.045(0.017)	$7.4x10^{-3}$	--++

Supplementary Table 2 (cont'd). Association signals for later expressive vocabulary (24-30 months, $p \leq 10^{-4}$)

SNP	E/A	Chr	Pos[a]	Discovery(N=6299) EAF	Beta(SE)[b]	p[b]	Meta-analysis(N=10819) EAF	Beta(SE)	p	Dir
rs2430894	A/G	18	52,568,589	0.7	-0.089(0.02)	1.2×10^{-5}	0.7	-0.035(0.015)	1.9×10^{-2}	++-+
rs9962179	T/C	18	54,221,584	0.68	-0.077(0.019)	6.5×10^{-5}	0.68	-0.059(0.015)	5.8×10^{-5}	-+--
rs344575	T/C	19	6,607,497	0.88	0.16(0.039)	5.2×10^{-5}	0.89	0.11(0.029)	8.4×10^{-5}	+++-
rs4816169	A/C	20	972,652	0.68	0.12(0.029)	5.2×10^{-5}	0.67	0.089(0.025)	2.9×10^{-4}	-++? [c]
rs6030120	T/C	20	40,367,121	0.77	-0.083(0.021)	9.8×10^{-5}	0.77	-0.051(0.016)	1.7×10^{-3}	+---
rs11907293	T/G	20	54,867,252	0.02	-0.45(0.1)	1.3×10^{-5}	0.02	-0.29(0.088)	1.1×10^{-3}	+--? [c]

Genome-wide screen of expressive vocabulary scores between 24-30 months of age. Discovery analysis was conducted in ALSPAC and independent signals ($p \leq 1 \times 10^{-4}$) were followed up in Raine (N=981), TEDS (N=1727) and GenR (N=1812; **Supplementary Data 1**). Combined results are from inverse-variance fixed effect meta-analysis. Beta coefficients represent the change in rank-transformed score (adjusted for sex, age, age squared and the most significant principal components in each cohort) per effect allele from weighted linear regression of the score on allele dosage (MACH2QTL/SNPTEST). Signals based on more than one missing cohort were excluded. E – Effect allele, A – Alternative allele, Chr – Chromosome, Pos – Position, EAF – Effect allele frequency, Dir – Direction of the genetic effect in the discovery and follow-up cohort; a - hg18, b - Genomic-control corrected, c - Available in ALSPAC, Raine and GenR only (Total N=9092)

Supplementary Table 3. Genotyping characteristics for lead association signals (15-18 months)

SNP		ALSPAC (Discovery)	GenR (Follow-up)	Raine (Follow-up)	TEDS (Follow-up)	NFBC1966 (Sensitivity)
**rs7642482 (G,A)**	EAF (G)	0.18	0.19	0.18	0.17	0.15
	Imputation quality (MACH R^2 / PROPERINFO)	R^2 = 0.95	R^2= 0.96	R^2=0.97	PROPERINFO=0.93	PROPERINFO=0.97
**rs10734234 (T,C)**	EAF (T)	0.90	0.90	0.90	0.88	0.91
	Imputation quality (MACH R^2 / PROPERINFO)	R^2= 0.76	R^2= 0.75	R^2= 0.72	PROPERINFO=0.77	PROPERINFO=0.53
**rs11176749 (T,A)**	EAF (T)	0.11	0.11	0.12	0.11	0.13
	Imputation quality (MACH R^2 / PROPERINFO)	R^2= 1.00	R^2= 1.00	R^2=1.00	PROPERINFO=0.91	PROPERINFO=0.99
**rs1654584(G,T)**[a]	EAF (G)	0.23	0.23	0.22	-	0.22
	Imputation quality (MACH R^2 / PROPERINFO)	R^2= 1.00	R^2= 1.00	R^2=1.00	-	PROPERINFO=0.98

EAF – Effect allele frequency; All SNPs were imputed, a - Available in ALSPAC, Raine and GenR only

Supplementary Table 4. Association analysis at rs10734234

**Phase**	**Age (m)**	**I/G**	**E/A**	**EAF**	**Beta(SE)**	***p***
Early	15	0	T/C	0.88	-0.11(0.027)	$1.8x10^{-5}$
	15	1	T/C	0.90	-0.14(0.032)	$5.7x10^{-6}$
Late	24	0	T/C	0.88	-0.09(0.027)	$1.0x10^{-3}$
	24	1	T/C	0.90	-0.11(0.034)	$5.0x10^{-4}$

Association analysis comparing directly genotyped versus imputed SNP data (N=8,058) in the discovery cohort (ALSPAC). Beta coefficients represent the change in rank-transformed score (adjusted for sex, age, age squared and the most significant principal components in ALSPAC) per effect allele from linear regression of the score on allele dosage. I/G – Imputed (1)/ directly genotyped (0)SNP, E – Effect allele, A – Alternative allele, EAF – Effect allele frequency, m - months

Supplementary Table 5. Adjustment of lead association signals (15-18 months) for potential covariates

			Discovery						Follow-up					Meta-analysis			
**SNP**	**E/A**	**Model**	**N**	**EAF**	**Beta(SE)**	***p***	**R^2 (%)**	**N**	**EAF**	**Beta(SE)**	***p***	**R^2(%)**	**N**	**Beta(SE)**	***p***	**p_{het}**
Covariate: Gestational age																
rs7642482	G,A	baseline	6851	0.18	-0.11(0.022)	9.6×10^{-7}	0.34	2038	0.19	-0.11(0.040)	0.0044	0.35	8889	-0.11(0.019)	1.4×10^{-8}	0.91
rs7642482	G,A	adj		0.18	-0.11(0.022)	3.6×10^{-7}	0.36		0.19	-0.12(0.040)	0.0032	0.38		-0.11(0.019)	4.0×10^{-9}	0.91
rs10734234	T,C	baseline		0.90	-0.14(0.032)	9.7×10^{-6}	0.27		0.90	-0.17(0.058)	0.0045	0.35		-0.15(0.028)	1.5×10^{-7}	0.71
rs10734234	T,C	adj		0.90	-0.14(0.032)	9.0×10^{-6}	0.27		0.90	-0.17(0.059)	0.0046	0.35		-0.15(0.028)	1.4×10^{-7}	0.72
rs11176749	T,A	baseline		0.11	-0.12(0.027)	2.0×10^{-5}	0.25		0.11	-0.13(0.050)	0.010	0.27		-0.12(0.024)	6.6×10^{-7}	0.83
rs11176749	T,A	adj		0.11	-0.12(0.027)	8.0×10^{-6}	0.28		0.11	-0.13(0.050)	0.0081	0.29		-0.12(0.024)	2.1×10^{-7}	0.85
rs1654584	G,T	baseline		0.23	-0.081(0.02)	6.8×10^{-5}	0.22		0.23	-0.13(0.038)	9.1×10^{-4}	0.49		-0.091(0.018)	3.6×10^{-7}	0.30
rs1654584	G,T	adj		0.23	-0.083(0.02)	5.1×10^{-5}	0.22		0.23	-0.13(0.038)	6.1×10^{-4}	0.53		-0.093(0.018)	2.0×10^{-7}	0.28
Covariate: Maternal education																
rs7642482	G,A	baseline	6681	0.18	-0.11(0.022)	5.8×10^{-7}	0.36	2015	0.19	-0.11(0.040)	0.0058	0.33	8696	-0.11(0.020)	1.1×10^{-8}	0.99
rs7642482	G,A	adj		0.18	-0.11(0.023)	4.7×10^{-7}	0.36		0.19	-0.11(0.040)	0.0077	0.30		-0.11(0.020)	1.2×10^{-8}	0.90
rs10734234	T,C	baseline		0.90	-0.14(0.032)	1.2×10^{-5}	0.27		0.90	-0.17(0.059)	0.0048	0.35		-0.15(0.028)	2.0×10^{-7}	0.71
rs10734234	T,C	adj		0.90	-0.14(0.032)	1.2×10^{-5}	0.27		0.90	-0.17(0.059)	0.0039	0.36		-0.15(0.028)	1.6×10^{-7}	0.68
rs11176749	T,A	baseline		0.11	-0.12(0.027)	1.6×10^{-5}	0.26		0.11	-0.13(0.050)	0.012	0.27		-0.12(0.024)	5.6×10^{-7}	0.88
rs11176749	T,A	adj		0.11	-0.12(0.027)	1.6×10^{-5}	0.26		0.11	-0.12(0.050)	0.012	0.26		-0.12(0.024)	6.2×10^{-7}	0.91
rs1654584	G,T	baseline		0.23	-0.08(0.021)	0.00011	0.21		0.23	-0.12(0.038)	0.0022	0.42		-0.089(0.018)	8.8×10^{-7}	0.36
rs1654584	G,T	adj		0.23	-0.08(0.021)	0.00011	0.21		0.23	-0.12(0.038)	0.0022	0.42		-0.089(0.018)	8.9×10^{-7}	0.36

Adjustment of lead signals for potential covariates are shown for the discovery (ALSPAC) and follow-up (GenR) cohort, and inverse-variance fixed effect meta-analysis. Beta coefficients represent the change in rank-transformed score per effect allele from linear regression of the score on allele dosage (R/Stata/SPSS software). Covariate details are described in **Supplementary Data 1**. Baseline - Baseline models: Expressive vocabulary scores were adjusted for sex, age, age squared and the most significant principal components in each cohort before rank-transformation, Adj - Adjusted models: As baseline models with additional adjustment for the covariate; E – Effect allele, A – Alternative allele, EAF – Effect allele frequency, p_{het} – Heterogeneity *p*-value based on Cochran's Q-test, R^2 – Adjusted regression R^2 in %

Supplementary Table 6. Association between lead association signals (15-18 months) and potential covariates

		Discovery			Follow-up			Meta-analysis			
Covariate: Gestational age											
**SNP**	**E/A**	**N**	**Beta(SE)**	***p***	**N**	**Beta(SE)**	***p***	**N**	**Beta(SE)**	***p***	**p_{het}**
rs7642482	G,A	7877	0.082(0.038)	0.028	2649	0.074(0.054)	0.17	10526	0.078(0.031)	0.0096	0.90
rs10734234	T,C		-0.0046(0.054)	0.93		0.0065(0.079)	0.93		-0.0011(0.045)	0.98	0.91
rs11176749	T,A		0.042(0.046)	0.36		0.068(0.067)	0.31		0.050(0.038)	0.18	0.74
rs1654584	G,T		0.012(0.034)	0.73		0.050(0.05)	0.32		0.024(0.028)	0.40	0.52
Covariate: Maternal education											
**SNP**	**E/A**	**N**	**OR(SE)**	***p***	**N**	**OR(SE)**	***p***	**N**	**OR(SE)**	***p***	**p_{het}**
rs7642482	G,A	7407	0.98(0.049)	0.75	2596	1.01(0.017)	0.49	10003	1.01(0.016)	0.59	0.60
rs10734234	T,C		0.96(0.068)	0.55		0.98(0.025)	0.32		0.97(0.023)	0.25	0.82
rs11176749	T,A		1.03(0.061)	0.63		1.01(0.021)	0.75		1.01(0.02)	0.64	0.73
rs1654584	G,T		0.98(0.044)	0.60		1.01(0.016)	0.36		1.01(0.015)	0.49	0.42

Association between potential covariates and lead signals for rank-transformed CDI expressive vocabulary scores between 15-18 months of age. Results are shown for the discovery (ALSPAC) and follow-up (GenR) cohort, and inverse-variance fixed effect meta-analysis. Beta coefficients represent the change in gestational age (weeks) per effect allele from linear regression of gestational age on allele dosage, adjusted for sex and the most significant principal components in each cohort. Odds ratios (OR) represent the odds of having lower compared to higher maternal education per effect allele from logistic regression of maternal education (low=1, high=0) on allele dosage, adjusted for the most significant principal components in each cohort. Covariate details are described in **Supplementary Data 1**. E – Effect allele, A – Alternative allele, EAF – Effect allele frequency, p_{het} – Heterogeneity *p*-value based on Cochran's Q-test

Supplementary Table 7. Association between lead association signals (15-18 months) and first single-word utterances (12 months)

**SNP**	**E/A**	**EAF**	**OR(SE)**	***p***
rs7642482	G/A	0.15	1.03(0.07)	0.68
rs10734234	T/C	0.91	1.08(0.13)	0.51
rs11176749	T/A	0.13	0.98(0.07)	0.76
rs1654584	G/T	0.22	0.96(0.06)	0.47

Expressive vocabulary was assessed as the number of words spoken at the age of 12 months In the NFBC1966. Odds ratios (OR) represent the odds of speaking one or more words compared with speaking no words per effect allele and were obtained from logistic regression of expressive vocabulary (1+ words (1) = 3856 children, 0 words (0) = 1113 children, N=4969) on allele dosage, adjusted for sex and the most significant principal components. E – Effect allele, A – Alternative allele, EAF – Effect allele frequency

Supplementary Table 8. Language-related cognitive outcomes in later childhood

SNP	Mean score(SD) untransformed	Mean age at measurement (SD) in years
Phonological memory	7.26(2.51)	8.63(0.3)
Verbal intelligence	107.81(16.75)	8.64(0.31)
Reading speed (Words read per minute)	105.51(12.47)	9.89(0.32)
Reading comprehension	100.4(11.83)	9.89(0.32)

Sample descriptives for language-related cognitive outcomes in later childhood measured in ALSPAC. Phonological memory was assessed with 'The Children's Test of Nonword Repetition'[3], verbal intelligence quotient scores (Verbal IQ) with the' Wechsler Intelligence Scale for Children'[4,5], and reading speed ('words read per minute') as well as reading comprehension with the Neale analysis of reading ability test[6]. SD – Standard deviation

Supplementary Table 9. Association between lead association signals (15-18 months) and language-related cognitive outcomes in later childhood

SNP	E/A	N	Beta(SE)	*p*
Phonological memory (8 years)				
rs7642482	G/A	5552	0.021(0.025)	0.39
rs10734234	T/C		0.011(0.036)	0.77
rs11176749	T/A		-0.034(0.030)	0.27
rs1654584	G/T		-0.018(0.023)	0.43
Verbal IQ (9 years)				
rs7642482	G/A	5540	-0.032(0.025)	0.20
rs10734234	T/C		-0.040(0.036)	0.26
rs11176749	T/A		-0.022(0.030)	0.48
rs1654584	G/T		-0.035(0.023)	0.12
Reading speed (Words read per min, 10 years)				
rs7642482	G/A	5275	-0.067(0.026)	0.0093
rs10734234	T/C		0.052(0.037)	0.16
rs11176749	T/A		-0.011(0.031)	0.72
rs1654584	G/T		-0.068(0.023)	0.0035
Reading comprehension (10 years)				
rs7642482	G/A	5287	-0.042(0.026)	0.10
rs10734234	T/C		0.004(0.042)	0.93
rs11176749	T/A		-0.012(0.031)	0.70
rs1654584	G/T		-0.051(0.023)	0.028

Sample descriptives for all cognitive measures are given in **Supplementary Table 8.** Beta coefficients represent the change in cognitive outcome (Z-standardised) per effect allele from linear regression of the cognitive outcome on allele dosage, adjusted for sex, the most significant principal components and age (except for age-normalised Verbal IQ scores). E – Effect allele, A – Alternative allele

Supplementary Table 10. Twin analysis of expressive vocabulary scores (24 months)

Twin intra-class correlations		ACE model Variance components (95% CI)		
		**A**	**C**	**E**
**MZ**	0.94; N = 1969	0.20	0.73	0.07
**DZ all**	0.84; N = 3764	(0.19;0.22)	(0.72;0.75)	(0.06;0.07)

Expressive vocabulary was assessed with the MCDI[7,8] at 24 months of age within TEDS. Twin analysis was conducted on 5,733 twin pairs using rank-transformed expressive vocabulary scores adjusted for age, age squared and sex. The best-fitting model (ACE) was chosen in comparison to a CE and AE model on the basis of model fit parameters (**Supplementary Table 11**).

MZ – Monozygotic twins, DZall – Dizygotic twins (male, female, opposite sex), N – Complete twin pairs,
A – Additive genetic influence, C – Shared environmental influence, E – Non-shared environmental influence

Supplementary Table 11. Model fit parameters for twin analysis

**Model**	**-2LL**	**df**	**AIC**	***p*(1)**	***p*(2)**
Saturated	24279.678	11504	1271.678	-	-
ACE*	24286.858	11507	1272.858	.07	-
CE	24749.167	11508	1733.167	<0.001	<0.001
AE	25972.380	11508	2956.380	<0.001	<0.001
E	32631.156	11509	9613.156	<0.001	<0.001

-2LL - Minus twice the log likelihood; df - Degrees of freedom; AIC - Akaike Information Criterion; *p*(1) - *p*-value of model fit compared to the saturated model; *p*(2) - *p*-value of model fit compared to the ACE model; * - Best-fitting model; A-Additive genetic influence; C-Shared environmental influence; E-Non-shared environmental influence.

Supplementary Table 12. Association between *ROBO1* signals and early expressive vocabulary (15 months)

SNP	E/A	Proxy	E/A	r^2	Chr	Pos[a]	EAF	Beta(SE)	*p*
rs12495133	A/C	rs1383407	C/T	0.97	3	78,908,171	0.42	-0.006(0.02)	0.73
rs331142	C/A	rs331146	C/T	1	3	78,917,283	0.24	0.001(0.02)	0.94
rs4535189	G/A	-	-	-	3	79,489,971	0.51	-0.02(0.017)	0.20
rs6803202	T/C	-	-	-	3	79,499,153	0.51	-0.02(0.017)	0.22

rs12495133 and rs331142 have been associated with reading disability[9], and rs6803202 and rs4535189 with performance on tasks of non-word repetition[10]. Association was studied using locally imputed genotypes on chromosome 3 (based on 1000 Genomes) in ALSPAC, as some variants (rs331142 and rs12495133) had no proxies in Hapmap 2 imputed data. Beta coefficients represent the change in rank-transformed score (adjusted for sex, age, age squared and the most significant principal components in each cohort) per effect allele from weighted linear regression of the score on allele dosage (MACH2QTL). Linkage disequilibrium with proxy SNPs is given in r^2. E – Effect allele, A – Alternative allele, Proxy – Proxy SNP, Chr – Chromosome, Pos – Position, EAF – Effect allele frequency; a – hg19

SUPPLEMENTARY NOTES

Supplementary Note 1. Cohort description and study-specific ethical approval

Avon Longitudinal Study of Parents and Children (ALSPAC)

Avon Longitudinal Study of Parents and Children (ALSPAC) is a population based longitudinal pregnancy-ascertained birth-cohort in the Bristol area of the UK. Specifically, recruitment sought to enrol all pregnant women with an estimated delivery date between 1st April 1991 and 31st December 1992, who were residents within three Health Districts of the former administrative county of Avon[11,12]. The initial cohort included 14,541 pregnancies and additional children eligible using the original enrolment definition were recruited up to the age of 18 years, increasing the total number of pregnancies to 15,247 (4.1% Non-White mothers). Information on the children from these pregnancies is available from questionnaires, clinical assessments, linkage to health and administrative records as well as biological samples including genetic and epigenetic information. Detailed information of all available data can be obtained online (http://www.bris.ac.uk/alspac/researchers/data-access/data-dictionary/). Ethical approval was obtained from the ALSPAC Law and Ethics Committee (IRB00003312) and the Local Research Ethics Committees, and written informed consent was provided by all parents.

Generation R (GenR)

The Generation R Study is a population-based prospective cohort from fetal life onwards in Rotterdam, the Netherlands, which has been described in detail elsewhere[13].Typically, enrolment took place in early pregnancy. All children were born between April 2002 and January 2006, forming a prenatally enrolled birth-cohort. The study was conducted in accordance with the guideline proposed in the World Medical Association Declaration of Helsinki and has been approved by the Medical Ethics Committee of the Erasmus Medical Centre, Rotterdam (numbers: MEC 198.782/2001/31 (prenatal) and MEC 217.595/2002/202 (postnatal)). Written informed consent was obtained from all participants.

Northern Finnish Birth Cohort 1966 (NFBC 1966)

The Northern Finland Birth Cohort 1966 (NFBC1966)[14] was recruited through maternity health centres and data were collected from women living in Finland's two northernmost provinces, Oulu and Lapland, with expected deliveries between 1st January to 31st December 1966 (n=12,055 mothers). A total of 12,231 babies were born from the pregnancies, of which 12,058 were live births babies and 173 were stillborn babies. Individuals born in NFBC1966 were found to be representative of all births in the area. All cohort members are Finns (white Caucasians), and less than 1% of these are Gypsies or Lapps. Birth outcomes were collected at delivery by trained medical staff and input into the medical records. The individuals were then followed-up with questionnaires from birth to ages at 1, 14 and clinical examination at 31 years, covering information on health, lifestyle and socio-economic indicators. Each participant or their parents gave written informed consent for the use of the data (Protocols approved by the Ethical Committee of the Northern Ostrobothnia Hospital District).

The Twins Early Development Study (TEDS)

The Twins Early Development Study (TEDS) is a large longitudinal sample of twins born in England and Wales between 1994 and 1996[15]. The focus of TEDS has been on cognitive and behavioural development, including difficulties in the context of normal development. TEDS began when multiple births were identified from birth records and the families were invited to take part in the study; 16,810 pairs of twins were originally enrolled in TEDS. More than 10,000 of these twin pairs remain enrolled in the study to date. DNA has been collected for more than 7,000 pairs, and genome-wide genotyping data for two million DNA markers are available for 3,500 individuals. The TEDS families have taken part in studies when the twins were aged 2, 3, 4, 7, 8, 9, 10, 12, 14, 16 and currently at 18 years of age. Ethical approval for each stage of TEDS has been obtained from the Institute of Psychiatry Ethics Committee (REC approval 05/Q0706/228), and informed consent was collected from the parents for each assessment.

Western Australian Pregnancy Cohort study (Raine)

The Western Australian Pregnancy Cohort study (Raine)[16] was started as a randomised controlled trial to evaluate the effects of repeated ultrasound in pregnant women in Perth, Western Australia. In total, 2,900 pregnant women were recruited between 1989 and 1991 prior to 18 weeks gestation at

the King Edward Memorial Hospital (Perth, Western Australia). Women were randomised to repeated ultrasound measurements at 18, 24, 28, 34 and 38 weeks gestation or to a single ultrasound assessment at 18 weeks. Children have been assessed at average ages of 1, 2, 3, 5, 8, 10, 14 and 17 and both height and weight were collected at each assessment. The study was conducted with appropriate institutional ethics approval (Ethics approval number for DNA collection and storage: EC03-14.7 and EC06-29), and written informed consent was obtained from mothers at all follow-ups and participants at the year 17 follow-up.

Supplementary Note 2. URLs

ALSPAC, http://www.bris.ac.uk/alspac/researchers/data-access/data-dictionary

Brainspan, http://www.brainspan.org

EAGLE, http://research.lunenfeld.ca/eagle

Golden Path Genome Browser, http://genome.ucsc.edu

GWAVA http://www.sanger.ac.uk/sanger/StatGen_Gwava

HaploReg, v2 http://www.broadinstitute.org/mammals/haploreg/haploreg.php

HapMap, http://hapmap.ncbi.nlm.nih.gov

LocusZoom, http://csg.sph.umich.edu/locuszoom

PLINK, http://pngu.mgh.harvard.edu/~purcell/plink

OMIM, http://www.ncbi.nlm.nih.gov/omim

seeQTL, http://www.bios.unc.edu/research/genomic_software/seeQTL

Supplementary Note 3. Consortium membership

Wellcome Trust Case Control Consortium 2 (WTCCC2)

Management Committee:

Peter Donnelly (Chair)[1,2], Ines Barroso (Deputy Chair)[3], Jenefer M Blackwell[4, 5], Elvira Bramon[6] , Matthew A Brown[7] , Juan P Casas[8] , Aiden Corvin[9],Panos Deloukas[3], Audrey Duncanson[10], Janusz Jankowski[11], Hugh S Markus[12], Christopher GMathew[13], Colin NA Palmer[14], Robert Plomin[15], Anna Rautanen[1], Stephen J Sawcer[16], Richard CTrembath[13], Ananth C Viswanathan[17], Nicholas W Wood[18]

Data and Analysis Group:

Chris C A Spencer[1], Gavin Band[1], Céline Bellenguez[1], Colin Freeman[1], Garrett Hellenthal[1], Eleni Giannoulatou[1], Matti Pirinen[1], Richard Pearson[1], Amy Strange[1], Zhan Su[1], Damjan Vukcevic[1], Peter Donnelly[1,2]

DNA, Genotyping, Data QC and Informatics Group:

Cordelia Langford[3], Sarah E Hunt[3], SarahEdkins[3], Rhian Gwilliam[3], Hannah Blackburn[3], Suzannah J Bumpstead[3], Serge Dronov[3],Matthew Gillman[3], Emma Gray[3], Naomi Hammond[3], Alagurevathi Jayakumar[3], Owen T McCann[3], Jennifer Liddle[3], Simon C Potter[3], Radhi Ravindrarajah[3], Michelle Ricketts[3], Matthew Waller[3], Paul Weston[3], Sara Widaa[3], Pamela Whittaker[3], Ines Barroso[3], Panos Deloukas[3]

Publications Committee:

Christopher G Mathew (Chair)[13], Jenefer M Blackwell[4,5], Matthew A Brown[7], Aiden Corvin[9], Chris C A Spencer[1]

WTCCC2 member affiliations:

1 Wellcome Trust Centre for Human Genetics, University of Oxford, Roosevelt Drive, OxfordOX3 7BN, UK

2 Department of Statistics, University of Oxford, Oxford OX1 3TG, UK

3 Wellcome Trust Sanger Institute, Wellcome Trust Genome Campus, Hinxton, Cambridge CB10 1SA, UK

4 Telethon Institute for Child Health Research, Centre for Child Health Research, University of Western Australia, 100 Roberts Road, Subiaco, Western Australia 6008

5 Cambridge Institutefor Medical Research, University of Cambridge School of Clinical Medicine, Cambridge CB20XY, UK

6 Department of Psychosis Studies, NIHR Biomedical Research Centre for MentalHealth at the Institute of Psychiatry, King's College London and The South London and Maudsley NHS Foundation Trust, Denmark Hill, London SE5 8AF, UK

7 University of Queensland Diamantina Institute, Brisbane, Queensland, Australia

8 Department of Epidemiology and Population Health, London School of Hygiene and Tropical Medicine, London WC1E 7HT and Department of Epidemiology and Public Health, University College London WC1E 6BT, UK

9 Neuropsychiatric Genetics Research Group, Institute of Molecular Medicine, Trinity CollegeDublin, Dublin 2, Eire

10 Molecular and Physiological Sciences, The Wellcome Trust, LondonNW1 2BE, UK

11 Department of Oncology, Old Road Campus, University of Oxford, Oxford OX37DQ, UK, Digestive Diseases Centre, Leicester Royal Infirmary, Leicester LE7 7HH, UK and Centre for Digestive Diseases, Queen Mary University of London, London E1 2AD, UK

12 Clinical Neurosciences, St George's University of London, London SW17 0RE, UK

13 King's College London Dept Medical and Molecular Genetics, King's Health Partners, Guy's Hospital,London SE1 9RT, UK

14 Biomedical Research Centre, Ninewells Hospital and Medical School,Dundee DD1 9SY, UK

15 King's College London Social, Genetic and Developmental PsychiatryCentre, Institute of Psychiatry, Denmark Hill, London SE5 8AF, UK

16 University of CambridgeDept Clinical Neurosciences, Addenbrooke's Hospital, Cambridge CB2 0QQ, UK

17 NIHR Biomedical Research Centre for Ophthalmology, Moorfields Eye Hospital NHS FoundationTrust and UCL Institute of Ophthalmology, London EC1V 2PD, UK

18 Department of Molecular Neuroscience, Institute of Neurology, Queen Square, London WC1N 3BG, UK

SUPPLEMENTARY REFERENCES

1. Fenson, L., Dale, P. & Reznic, S. *Technical Manual for the MacArthur Communicative Development Inventories*. (Developmental Psychology Laboratory, 1991).
2. Reznick, J. S. & Goldsmith, L. A multiple form word production checklist for assessing early language. *J Child Lang* **16,** 91–100 (1989).
3. Gathercole, S. E., Willis, C. S., Baddeley, A. D. & Emslie, H. The Children's Test of Nonword Repetition: a test of phonological working memory. *Memory* **2,** 103–127 (1994).
4. Wechsler, D., Golombok, J. & Rust, J. *WISC-IIIUK Wechsler Intelligence Scale for Children – UK Manual*. (The Psychological Corporation, 1992).
5. Adebamowo, C. *et al.* A United Kingdom population-based study of intellectual capacities in children with and without soiling, daytime wetting, and bed-wetting. *Pediatrics* **120,** e308–16 (2007).
6. Neale, M. D. *Neale analysis of reading ability*. (Nelson Thornes, 1997).
7. Fenson, L. *et al.* Short-Form Versions of the MacArthur Communicative Development Inventories. *Appl Psycholinguist* **21,** 95–116 (2000).
8. Dale, P. S., Dionne, G., Eley, T. C. & Plomin, R. Lexical and grammatical development: a behavioural genetic perspective. *J Child Lang* **27,** 619–642 (2000).
9. Tran, C. *et al.* Association of the ROBO1 gene with reading disabilities in a family-based analysis: Association of the ROBO1 gene. *Genes Brain Behav* **13,** 430–438 (2014).
10. Bates, T. C. *et al.* Genetic variance in a component of the language acquisition device: ROBO1 polymorphisms associated with phonological buffer deficits. *Behav Genet* **41,** 50–57 (2011).
11. Boyd, A. *et al.* Cohort Profile: The 'Children of the 90s'—the Index Offspring of the Avon Longitudinal Study of Parents and Children. *Int J Epidemiol* **42,** 111–27 (2013).
12. Fraser, A. *et al.* Cohort Profile: The Avon Longitudinal Study of Parents and Children: ALSPAC Mothers Cohort. *Int J Epidemiol.* **42,** 97–110 (2012).
13. Jaddoe, V. W. V. *et al.* The Generation R Study: design and cohort update 2012. *Eur J Epidemiol* **27,** 739–756 (2012).
14. Rantakallio, P. Groups at risk in low birth weight infants and perinatal mortality. *Acta Paediatrica Scandinavia* **193,** 1–71 (1969).
15. Haworth, C. M. A., Davis, O. S. P. & Plomin, R. Twins Early Development Study (TEDS): A Genetically Sensitive Investigation of Cognitive and Behavioral Development From Childhood to Young Adulthood. *Twin Res Hum Genet* **16,** 117–125 (2013).
16. Newnham, J. P., Evans, S. F., Michael, C. A., Stanley, F. J. & Landau, L. I. Effects of frequent ultrasound during pregnancy: a randomised controlled trial. *The Lancet* **342,** 887–891 (1993).
